# Function and design of the Nox1 system in vascular smooth muscle cells

**DOI:** 10.1186/1752-0509-7-20

**Published:** 2013-03-11

**Authors:** Weiwei Yin, Eberhard O Voit

**Affiliations:** 1 The Wallace H. Coulter Department of Biomedical Engineering, Georgia Institute of Technology, 313 Ferst Drive, Atlanta, GA 30332, USA; 2 Integrative BioSystems Institute, Georgia Institute of Technology, 313 Ferst Drive, Atlanta, GA 30332, USA

## Abstract

**Background:**

Recent studies have demonstrated that the activation of NADPH oxidase 1 (Nox1) plays an important role in the control of reactive oxygen species and their involvement in vascular physiology and pathophysiology. In order to function properly, Nox1 needs to be available in an optimal state, where it is ready to respond appropriately and efficiently to upstream signals. It must also be able to return quickly to this state as soon as the input signal disappears. While Nox1 activation has been discussed extensively in recent years, mechanisms for enzyme disassembly and proper subunit recovery have not received the same attention and therefore require investigation.

**Results:**

We study the Nox1 system in vascular smooth smucle cells and propose four potential disassembly mechanisms. The analysis consists primarily of large-scale Monte-Carlo simulations whose results are essentially independent of specific parameter values. The computational analysis shows that a specific profile of subunit concentrations is crucial for optimal functioning and responsiveness of the system to input signals. Specifically, free p47^phox^ and inactive Rac1 should be dominant under unstimulated resting conditions, and the proteolytic disassembly pathway should have a low flux, as it is relatively inefficient. The computational results also reveal that the optimal design of the three subunit recovery pathways depends on the intracellular settings of the pathway and that the response speeds of key reversible reactions within the pathway are of great importance.

**Conclusions:**

Our results provide a systematic basis for understanding the dynamics of Nox1 and yield novel insights into its crucially important disassembly mechanisms. The rigorous comparisons of the relative importance of four potential disassembly pathways demonstrate that disassembly via proteolysis is the least effective mechanism. The relative significance of the other three recovery pathways varies among different scenarios. It is greatly affected by the required response speed of the system and depends critically on appropriate flux balances between forward and reverse reactions. Our findings are predictive and pose novel hypotheses that should be validated with future experiments.

## Background

Reactive oxygen species (ROS) play crucial roles as signaling molecules in vascular physiology [1,2], and aberrations in their profiles or function can lead to a wide spectrum of diseases [3-8]. Under normal physiological conditions, ROS are produced in a controlled manner and found in low concentrations [9]. They exert much of their signaling functions by influencing the activities of specific redox-sensitive genes, proteins, and pathways [2,9]. Under pathological conditions, ROS production is often increased. This increase leads to an unbalanced redox state, which is generically referred to as “oxidative stress” [10] and often involves deleterious processes that can damage cell structures and negatively alter lipids, proteins, and DNA [11-13]. Indeed, extended periods of oxidative stress have been shown, both with experimental and clinical evidence, to associate with a wide variety of cardiovascular and metabolic diseases [14,15], including hypertension, endothelial inflammation, diabetes, and atherosclerosis.

Elevated levels of ROS in the vascular system are often the result of several contributing factors at the production and removal side, but they are generally associated with the activation of vascular NADPH oxidase (Nox) [16-18], which responds to extracellular stimuli [19,20]. Once the production of ROS is initially triggered through reactions catalyzed by Nox, ROS can propagate their own production by enhancing activities of other ROS sources [21-23], including intracellular iron uptake, xanthine oxidase, uncoupled endothelial nitric oxide synthase (eNOS), and damaged mitochondria. This type of propagation constitutes a feedforward mechanism that can lead to a vicious cycle of amplification and the maintenance of endogenous ROS in large quantities, which in turn contribute to pathological signaling. Because vascular NADPH oxidase is at the center of this deleterious process, it has become the subject of extensive investigation.

NADPH oxidase is a member of a family of enzymes that transfer electrons from NADPH to molecular oxygen [23], thus producing specific amounts of superoxide (O_2_^.-^), one of the major ROS in vessel walls. The Nox family consists of seven catalytic homologues, four of which (Nox1, Nox2, Nox4, and Nox5) are found in the vasculature [24]. These Nox subtypes present in distinct subcellular compartments, respond to different agonists, and often mediate specific cellular functions [19,20,24-26]. Among them, Nox1 has received the most attention mainly because it can be triggered by many physiological or pathological stimuli [24], such as angiotensin II (AngII), a hormone that activates Nox1 in vascular smooth muscle cells (VSMCs) [27,28], and platelet derived growth factor (PDGF) [28]. Many experimental studies have pointed out that Nox1 is clearly implicated in vascular pathologies [16,24], including AngII-induced hypertension and hypertrophy [29], serum-induced proliferation and PDGF-induced migration in VSMCs [30,31], abnormal vascular growth and inflammation [24], and atherosclerosis [17,32].

Under normal physiological conditions, Nox1 serves several beneficial purposes. Importantly, it is a crucial component in several signal transduction pathways [33], and extracellular activators, such as AngII [1] and PDGF [1], typically use Nox1-catalyzed ROS as specific intracellular signaling molecules to transduce information to downstream signals [34]. If Nox1 does not respond to these extracellular activators efficiently, the ROS it generates are unable to exert their proper signaling function. As a consequence, extracellular signaling information may be lost, along with the intracellular processes or events that were to be triggered. Thus, for proper functioning, Nox1 needs to be maintained in an optimal state, where it is ready to respond appropriately to upstream signals by producing enough ROS to transduce the signal. By the same token, as soon as the input signal disappears, Nox1 must quickly return to its optimal state, where ROS production is low and the machinery for generating new ROS is ready for new upstream signals. In this sense, one may define the optimal state and dynamics for Nox1 under normal physiological condition by two demands. First, Nox1 needs be responsive to input signals. And second, once an input signal ceases, Nox1 must return to its normal state as soon as possible.

The control of Nox1 activity is quite complex, as it is associated with the assembly and disassembly of several components, and in order to understand Nox1 and its regulatory function, it is necessary to discuss its components in some detail. Owing to the large body of experimental studies, we have substantial information about Nox1’s molecular composition, intracellular compartmentalization, activation mechanisms, and possible physiological functions [20,24,33]. Generally, Nox1 becomes functionally active when it is complexed with several cytosolic subunits and regulators, including an “organizer” [20], an “activator” [20], and the small GTPase Rac [35]. Both the organizer and the activator exist in two homologues, namely, p47^phox^ or NoxO1 [36,37], and p67^phox^ or NoxA1 [36,37], respectively. These homologues have different, cell-specific spatial distributions, and their regulatory mechanisms are somewhat different [38]. The specific identity of the Nox1 regulatory subunits is not completely known in all vascular cells. A notable exception is the case of VSMCs, where Nox1 is complexed with the activator NoxA1 [39,40] and the organizer p47^phox^[16,40,41]. In this paper, we will specifically focus on this very pertinent cell type.

While we have learned much about the assembly and activation of Nox1, the equally important disassembly processes have received much less attention. This discrepancy might be due to limitations posed by the current experimental repertoire, as the dynamics of proteins moving within a complex pathway is difficult to assess. For instance, it is still unclear whether the active Nox1 complex disassociates into individual subunits or simply degrades via proteolysis once it is no longer needed. It is also unknown in which fashion the disassociation happens and, in fact, whether the order of disassembly is of any importance.

**Figure 1:**
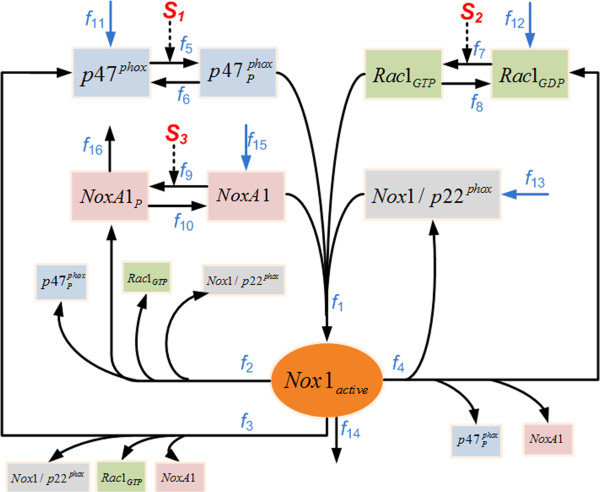
**Schematic representation of the Nox1 assembly-disassembly system in VSMCs.** Note the subtle differences among the three recycling pathways, which lead to differently phosphorylated or unphosphorylated products. For instance, only the pathway marked *f*_3_ directly produces the unphosphorylated form of p47^phox^ and only *f*_4_ leads directly to Rac1_GDP_.

Although the specific disassembly mechanisms of Nox1 in VSMCs are not known, studies of similar disassociation pathways for relevant Nox homolgues, or in other types of cells, have shed some light on possible mechanisms. For instance, using semi-recombinant and fully purified systems involving Nox2, membrane-associated guanine activating proteins (GAPs) were experimentally shown to be able to interact with the assembled oxidase complex and thereby to decrease its enzymatic activity by accelerating the hydrolysis of Rac1-bounded GTP [42]. This study suggests that the deactivation of GTP-bound Rac1 into the GDP-bound form (corresponding to the pathway marked with *f*_4_ in Figure 1) might be a possible disassembly pathway for Nox2 or its closest homologue Nox1. Addtionally, using Nox2 from neutrophiles, experiments with different activators (*e.g.*, kinases) and inhibitors (*e.g.*, phosphatases) have led to the suggestion that assembled p47^phox^ might be dephosphorylated, which would result in the deactivation of the active complex [43]. This finding points to another potential disassembly pathway for Nox1 (corresponding to the pathway marked with *f*_3_ in Figure 1). Just recently, new insights were obtained from using human embryonic kidney 293 (HEK293) cells that were transfected with Nox1 and other subunits. In these experiments, phosphorylation of multiple sites on NoxA1 was shown to have the capability of weakening the binding of NoxA1 to Nox1 and thereby of down-regulating the constitutive activity of Nox1 [44]. This result implicates phosphorylation of NoxA1 as a potential disassembly mechanism of Nox1 (corresponding to the pathway marked with *f*_2_ in Figure 1).

Based on the current understanding of the dynamics of Nox1 in VSMCs, as well as relevant knowledge of its closest homologue, Nox2, in other cells, we propose a pathway system for Nox1 assembly and disassembly as it is shown in Figure 1. The proposed disassembly system contains four pathways, namely, one direct degradation pathway via proteolysis (marked with *f*_14_ in Figure 1) and three independent disassociation pathways (marked as *f*_2_, *f*_3_, and *f*_4_), which differ in a subtle manner: *f*_2_ is the only pathway that directly recoups the subunit NoxA1p, *f*_3_ is the only pathway that directly recoups p47^phox^, and *f*_4_ is the only pathway that directly leads to Rac1_GDP_. While these individual pathways have not been experimentally verified specifically in VSMCs, they have been reported to play important roles in the disassembly of active enzymes for Nox1 or Nox2 in other types of cells [42-44], and are therefore reasonable candidates for the so-far ill-characterized disassembly of Nox1.

Given the key roles of active and inactive Nox1 in normal physiology and in pathophysiology, it is necessary to improve our understanding of Nox1 regulation with respect to both, assembly and disassembly. However, developing deeper insights poses a problem, as subtle differences in the rates of the various assembly and disassembly steps can have consequences that we are unable to quantify and interpret with intuition alone. We are therefore proposing in this work a computational modeling approach that allows us to answer questions about the complex dynamics of Nox1. Such questions have not been answered experimentally, and are in fact difficult to assess with purely experimental means. They include the following: Are the four proposed disassembly pathways equally important for the effective reestablishment of an appropriate profile of Nox1 subunits? Or is one, or are several of the pathways, more effective than others? Do any of the pathways dominate the disassembly process? How does the importance of each pathway change with particular cellular demands as well as intracellular factors, such as initial protein concentrations, required response speeds, and the net directions of the involved reactions?

These types of questions are difficult to address with experiments, but we will demonstrate here that we can pose and at least partially answer them with mathematical and computational means. Indeed, the mathematical model we develop here not only integrates all available biological information and addresses specific questions, but also answers a number of generic questions that target the principles governing the natural design and dynamics of the Nox1 system. They are of the type: “Why is this system organized in the fashion we see it and not in an alternative fashion that appears to be just as reasonable”? To achieve this level of generality, we will construct our models in a fashion that is only very mildly dependent on parameter values. Expressed differently, we will develop an entire ensemble of models and distill those with superior features. The initial target is the special case of Nox1 in VSMCs, but features of this representative template are likely to have relevance for other members of the Nox family as well.

## Results

### Importance of fine-tuned availability of Nox1 subunits

Before we discuss the details of our modelling results, it is beneficial to ascertain that a well-controlled disassembly strategy of Nox1 is indeed important and not at all random. In order to respond to extracellular activators like AngII and PDGF effectively, Nox1 needs to reside in a state that is prepared to receive signals and act on the ROS system in an effective and appropriate manner. We call this state the “Ready-to-Respond State” or “RtRS.” As soon as an input signal has been processed, Nox1 needs to return quickly to RtRS, where ROS production is low and the machinery for generating new ROS is ready for new upstream signals. Thus, RtRS and the dynamics with which Nox1 returns to this state must satisfy two demands, as discussed before. Nox1 needs to be maximally responsive to input signals and, once these cease, return to RtRS as soon as possible.

While many factors affect the performance of Nox1, the distribution of initial subunit concentrations is of particular importance for RtRS to be effective. The impact of such a profile can be demonstrated most lucidly with a thought experiment that makes extreme assumptions: Suppose that only two subunits (p47^phox^ and Rac1) were involved in the activation of Nox1 and that they were either totally absent (coded as “0”) or maximally available (coded as “1”), which simplifies the discussion to four possible states (Table 1). Let’s furthermore assume that Nox1 can respond to two possible signals (*S*_1_ and *S*_2_) and assess its responsiveness to arbitrary combinations of incoming signal trains of *S*_1_, *S*_2_, or *S*_1_ &*S*_2_. Table 1 shows that the responsiveness of this simplified system is directly dependent on its resting state, and that the response repertoire is severely limited, no matter in what state Nox1 resides. In reality, the states are somewhere between 0 and 1, but the simplified examples already indicate that their distribution is important. Similar thought experiments may be constructed if three subunits are involved in the Nox1 dynamics.

**Table 1:** Simplified examples illustrating the need for a proper distribution of Nox1 subunits

***Subunit distribution in RtRS***	***Responsiveness to representative signals***
[p47^phox^] = 0; [Rac1_GDP_] = 0;	No response to *S*_1_, *S*_2_, or *S*_1_ &*S*_2_
[p47^phox^] = 0; [Rac1_GDP_] = 1;	Response to *S*_2_, and *S*_1 _&*S*_2_, but no response to *S*_1_
[p47^phox^] = 1; [Rac1_GDP_] = 0;	Response to *S*_1_, and *S*_1_ &*S*_2_, but no response to *S*_2_
[p47^phox^] = 1; [Rac1_GDP_] = 1;	Response only to *S*_1_ &*S*_2_, but not to *S*_1_ or *S*_2_

A second factor influencing the responsiveness of Nox1 at RtRS is the relative speed of the rates in reversible reactions, namely the phosphorylation and dephosphorylation of p47^phox^, Rac1_GDP_, and NoxA1. Again, a thought experiment demonstrates the effects of such rates most easily. Suppose the phosphorylation of p47^phox^ is extremely slow, while dephosphorylation is very fast in comparison, so that p47^phox^ will essentially always be dephosphorylated. An immediate consequence is that no signals of type *S*_1_ have the capacity to trigger the system, because any newly formed phosphorylated p47^phox^ is rapidly deactivated and essentially no additional active p47^phox^ is formed (see Figure 1). In the opposite case, if the phosphorylation of p47^phox^ is fast, while dephosphorylation is very slow in comparison, any free p47^phox^ is immediately activated. Thus, even without incoming signals of type *S*_1_, any further addition of *S*_1_ will have little effect on this process, and the Nox1 system is again not responsive to *S*_1_. Similar arguments hold for the other two reversible reactions.

As a conclusion from these thought experiments, optimality of Nox1’s RtRS critically depends on the material distribution of subunits and on a fine-tuned balance between the rates in the reversible reactions of the system. Indeed, these two aspects are directly correlated with each other.

The computational results fall into two classes. The first class evaluates the relative importance of the four proposed disassembly mechanisms of Nox1 against two objective criteria of functional effectiveness (see details in the subsequent section) and investigates the relationship between the initial protein concentrations and the system’s effectiveness. Based on the findings in the first set of analyses, the second class of simulations further explores the functional importance of the relevant recovery pathways and their relationships to details of the intracellular pathway settings, such as the relative magnitudes of different flux rates and balances between forward and reverse reactions.

### Strategy of analysis and criteria of functional effectiveness

The Nox1 disassembly system is too complex to permit a comprehensive algebraic analysis of its design features, as it is possible in much simpler systems [45]. Instead, we employ a large-scale Monte-Carlo simulation approach where a model of the system is assessed thousands of times with parameter values that are randomly chosen from a large parameter space. Only those simulation results are retained that satisfy two objective criteria of functional effectiveness. The first criterion characterizes how efficiently the system responds to signals and how fast this response can be mounted. The rationale for this criterion is the presumed advantage for Nox1 to reside in an optimized state as much as possible, thereby ensuring maximal responsiveness to input signals with a minimal response time. The combination of these two aspects becomes particularly important when the system receives several signals within a relatively short time span: If the system is optimized to be launched most effectively from its nominal state, then the system should return to this state as quickly as possible after a signal has been transduced.

The second criterion reflects semi-quantitative observations from documented lab experiments that pertain to ROS production after 30 minutes in AngII [26,27,46] and to application of the protein kinase C (PKC) activator phorbol-12-myristate-13-acetate (PMA) [16] to stimulate VSMCs. Specifically, the following three observations constitute of the second criterion that an admissible Nox1 model instantiation has to satisfy. (1) After 4 hours of AngII treatment, the total protein of Nox1 subunits, including both Nox1/p22^phox^ (*X*_2_) and Nox1_active_(*X*_1_), is not significantly changed [26]. (2) After 30 minutes of AngII treatment, the system reaches a steady state or quasi-steady state with up-regulated Nox1_active_ (*X*_1_) [27,46]. (3) After 30 minutes of PMA treatment, Nox1_active_ (*X*_1_) activity is up-regulated since ROS production is increased [16].

### Assessment of Nox1 disassembly mechanisms according to the first criterion

In order to evaluate the system’s responsiveness to different signals and signal combinations, we investigate four distinct sets of input signals, namely individual signals *S*_1_, *S*_2_, or *S*_3_, as well as simultaneous *S*_1_ and *S*_2_ signals. Among these, three signal sets have been experimentally shown to possess the capacity of activating Nox1 in VSMCs [16,27]. The exception, and thus a prediction of the model, is the response to an individual *S*_3_ signal, which is likely to inhibit Nox1 activity, although this has not been directly verified yet in VSMCs.

A factor that could possibly affect the system’s responsiveness is the set of initial concentrations of the system components, especially for the cytosolic subunits p47^phox^, Rac1, and NoxA1. Indeed, two questions that need to be investigated are whether their active or inactive forms are dominant under resting conditions, and how different distributions between these states affect the system’s responsiveness to input signals. Because no experimental information is available to address these questions, we explore a large space of possible combinations for initial distributions of the cytosolic components. In order to facilitate this comparative analysis, we assume that the sum of each quantity in its active plus its inactive form is constant, with an arbitrary default value of 100. The initial quantity of a component in active form is assigned by sampling a truncated Gaussian distribution with either a higher mean or a lower mean, as formulated in Equation (1).

(1) $$\begin{array}{l} X_{i}\in^{\prime \prime }0^{\prime \prime }:\left\{ \begin{array}{ll} X_{i}=5, & X_{i}\leq 5\\ X_{i}\in N\left(25,5\right), & 5<X_{i}\leq 50\\ X_{i}=50, & X_{i}>50 \end{array}\right.\;,\;\\ X_{i}\in^{\prime \prime }1^{\prime \prime }:\left\{ \begin{array}{ll} X_{i}=50, & X_{i}\leq 50\\ X_{i}\in N\left(75,5\right), & 50<X_{i}\leq 95\\ X_{i}=95, & X_{i}>95 \end{array}\right. \end{array}$$

Here, “0” and “1” represent two truncated Gaussian distributions with a lower and higher mean, respectively. This strategy yields a total of 8 combinations of initial conditions (Table 2). For instance, index 4 in Table 2 codes for the vector [0 1 1] and represents the specific situation where the states of free p47^phox^, Rac1_GTP_, and phosphorylated NoxA1 are initially dominant.

**Table 2:** Combinations of initial conditions for the three cytosolic subunits contributing to Nox1 assembly

**Index**	**p47**^**phox**^_**P **_**(*X***_***4***_**)**	**Rac1**_**GTP **_**(*X***_***6***_**)**	**NoxA1**_**P **_**(*X***_***8***_**)**
1	0^a^	0	0
2	0	0	1
3	0	1	0
4	0	1	1
5	1	0	0
6	1	0	1
7	1	1	0
8	1	1	1

^*a*^ “0” represents a truncated Gaussian distribution with a lower mean, while “1” represents a truncated Gaussian distribution with a higher mean, as described in the Text.

Once the initial conditions and input signals are specified, the independent fluxes *f*_2*SS*_, *f*_3*SS*_, *f*_4*SS*_, *f*_6*SS*_, *f*_8*SS*_, *f*_9*SS*_ (or *f*_10*SS*_), *f*_14*SS*_, and *f*_16*SS*_ are randomly sampled between 1 and 100 with the same uniform distribution *U*(0, 2) in log_10_ space (see details in Methods regarding the choices of specific ranges), which ensures an appropriate spread of probabilities, and the system’s responsiveness is evaluated by the new steady state of Nox1_active_ (*X*_1_) under different input signals. Intriguingly, the averages of the new steady state of *X*_1_ are essentially unchanged among any of the four sets of input signals and for all combinations of initial conditions (see Additional file 1: Figure S2).

**Figure 2:**
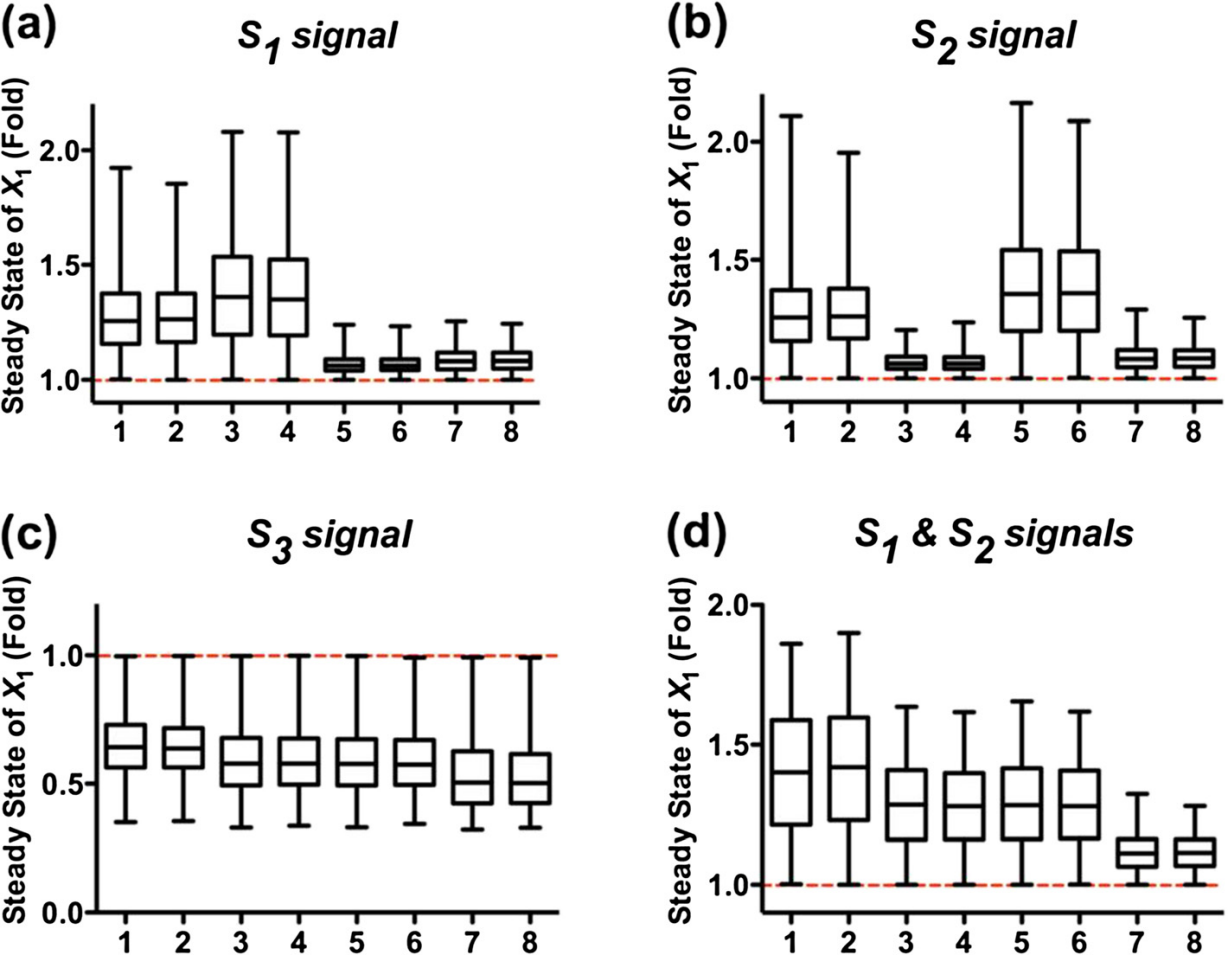
**Monte-Carlo simulation results for evaluating the functional effectiveness of the Nox1 disassembly system against generic criteria of responsiveness.** The four panels show results of the system receiving: (**a**) signal *S*_1_; (**b**) signal *S*_2_; (**c**) signal *S*_3_; (**d**) both signals, *S*_1_ and *S*_2_. All independent fluxes are sampled from *U*(0, 2) in log_10_ space except for *f*_14SS_, which equals zero. Simultion results are shown by box-and-whisker (min-to-max) plots. The dashed red lines indicate the steady state levels of *X*_1_ under control conditions, which are always set as “1”. Numbers along the *x*-axis ccorrespond to indices in Table 2 and represent different initial distributions. The sampling size for each bar is 2,000 points. Details of simulation settings are given in (Additional file 1: Table S1).

The situation is different when the flux through proteolysis (*f*_14*SS*_) is varied. If the numerical sampling range for this flux is gradually decreased, the system begins to mount responses to input signals (data not shown), given as the averages of new steady states of *X*_1_, that gradually increase or decrease under different input signals. An illustration is given for the extreme case of *f*_14*SS*_ = 0 (Figure 2), but very similar system responses are obtained for small flux values (results not shown). As a consequence of the absence (or small magnitude) of this flux, the system has the most effective responses as the steady state of *X*_1_ is enabled to increase or decrease greatly in response to different input signals. Expressed differently, a relatively low flux *f*_14*SS*_, in comparison to the other fluxes, “sensitizes” *X*_1_, thus indicating the importance of the relative low magnitude of *f*_14*SS*_ for the system’s responsiveness to input signals.

The same simulation results demonstrate that different combinations of initial conditions (indicated by different numbers along the *x*-axis in Figure 2) affect the system performance and lead to distinct response patterns in the presence of different input signals. When *S*_1_ signal is present (Figure 2 (a)), combinations 1 to 4 have an average of the new steady state of *X*_1_ that is much higher than 1. Thus, these combinations exhibit more effective responses than combinations 5 to 8, which have an average of the new steady state of *X*_1_ just merely above 1.

Detailed comparisons of these four combinations (1, 2, 3, 4) from Table 2 reveal one commonality: for all four combinations, p47^phox^ is initially dominant in the inactive form. This finding suggests that the dominance of free p47^phox^ is critial for the system’s responsiveness with respect to *S*_1_ signals. Analogous findings hold for *S*_2_ signals (Figure 2b). Here, combinations 1, 2, 5, and 6 exhibit better performance than other alternatives, indicating the necessity of Rac1 being initially dominant in its inactive form for an effective response to signal *S*_2_.

Taken together, keeping p47^phox^ and Rac1 dominant in their inactive states appears to be a superior strategy, when *S*_1_ and *S*_2_ signals are encountered. This inference is further supported by simulations with both signals, *S*_1_ and *S*_2_ (Figure 2d). In this case, combinations 7 and 8, which correspond to the situations that both p47^phox^ and Rac1 are dominant in their active forms, exhibit the poorest performance compared to other combinations. In contrast, combinations 1 and 2, with p47^phox^ and Rac1 dominant in their inactive states, exhibit the most effective responses, suggesting that this initial subunit distribution is a superior design among all alternatives. This computational insight correlates well with the previous, and so far unexplained, experimental observation that free p47^phox^ and inactive Rac1 are found as the dominant quantities under normal physiological conditions [41,47,48]. At the same time, this experimental support lends credence to the relevance of the first criterion.

With respect to signal *S*_3_, all combinations exhibit a similar performance (Figure 2c). Therefore, these results cannot be used to distinguish the effectiveness of combinations 1 and 2.

### Assessment of Nox1 disassembly mechanisms according to the second criterion

Similar to the analysis in the previous section, input signals and initial conditions need to be specified before simulations can be performed. Here the situation is different, because the second criterion, corresponding to three experimetnal conditions, is associated with semi-quantitative experimental observations, which allow us to replace the sampling scheme with direct experimental information about input signals and initial conditions. Details of the correspondence between the experimental, extracellular treatments and the numerical characteristics of the input signals and initial conditions for the model are summarized in Additional file 1: Table S2.

While valuable, the experimental data are not sufficient to determine specific parameter values, and it is still necessary to sample the independent fluxes. They are randomly selected between 0.01 and 100 from the uniform distribution *U*(−2, 2) in log_10_ space, and each sampled set is screened against three experimental conditions described before. The wide range is expected to cover all pertinent combinations of fluxes, and therefore appears to be adequate for the initial sampling. Ratios beyond this range would result in either very fast or very slow reactions, which would ultimately render a dependent variable static.

**Figure 3:**
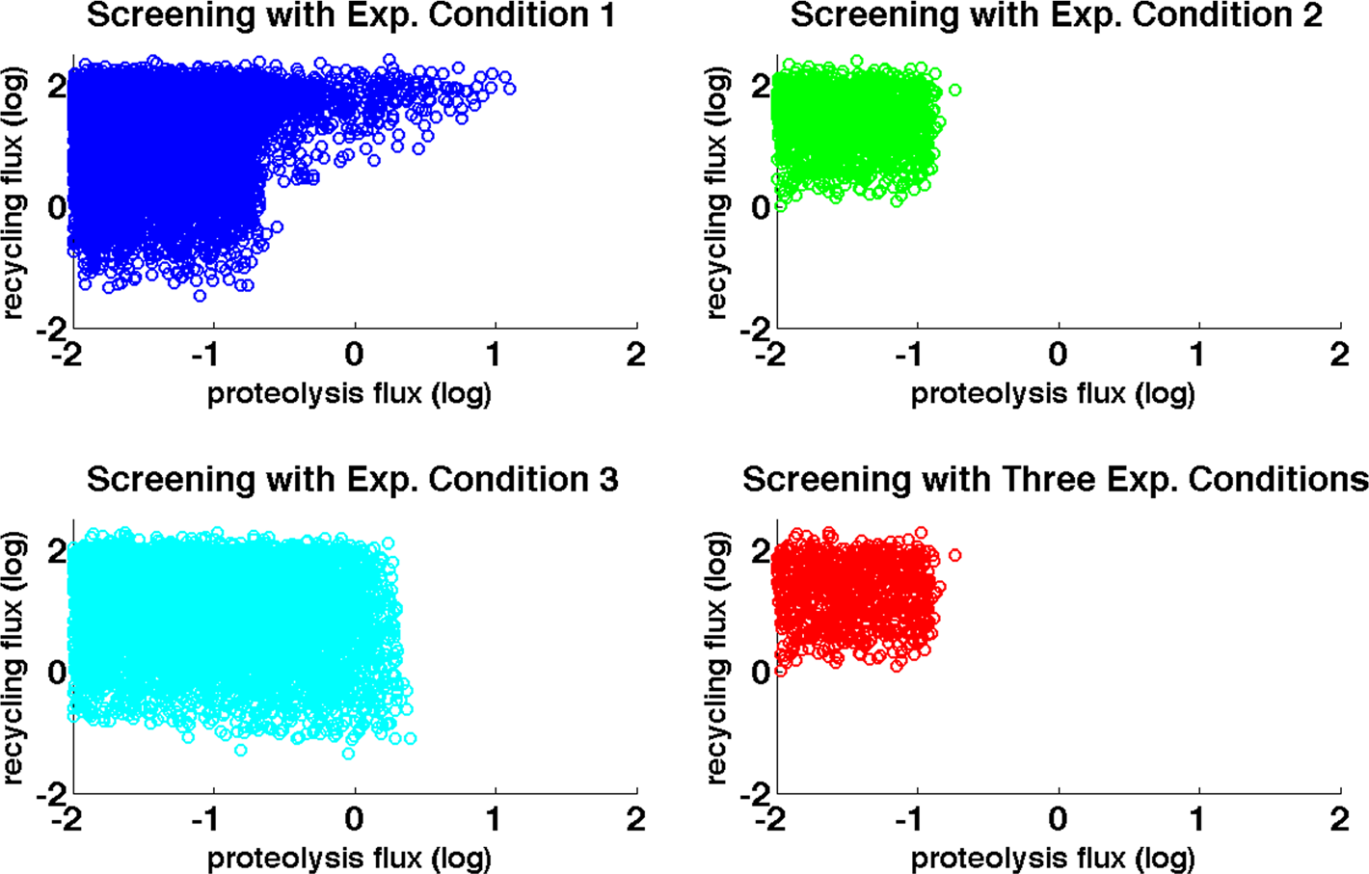
**Monte-Carlo simulation results for evaluating the functional effectiveness of the Nox1 disassembly system against three experimental observations.** Top-left: 6964 points (corresponding to combinations of proteolytic and recycling flux magnitudes) are retained after screening against the first experimental observation. Top-right: 1476 points are retained after screening against the second experimental observation; Bottom-left: 5460 points are retained after screening against the third experimental observation; Bottom-right: 825 points are retained after screening against all three experimental observations. See text for further details on specific experimental conditions.

With the settings thus described, we can examine combinations of flux rates of the proteolytic pathway (*f*_14SS_) and the collection of recycling pathways (summations of *f*_2SS_, *f*_3SS_, and *f*_4SS_) by screening preliminarily sampled points (see Additional file 1: Figure S3) against the different experimentally derived conditions (Figure 3). We begin by testing flux combinations against the first experimental finding of a stable Nox1 protein level that is sustained for 4 hours of AngII treatment. Interestingly, forcing all admissible flux combinations to match this criterion filters out almost all sampling points located in the lower-right region of the plot, where the proteolytic flux is large and the recycling flux is small. In other words, the combination of a large proteolytic flux and a small recycling flux is inefficient, and this finding directly and independently confirms the results presented before. Next we screen the flux combinations against the second experimental finding of an up-regulated (quasi-) steady state of Nox1_active_ (*X*_1_) at 30 minutes of AngII treatment. This screen filters out different combinations of fluxes. The remaining, successful combinations locate mainly in the upper-left region where the proteolytic flux is smaller than 1 while the recycling flux is larger than 1. Finally, filtering the flux combinations against the third experimental finding, namely up-regulated Nox1_active_ (*X*_1_) under PMA treatment, effectively excludes almost all combinations that are located to the very right, where the numerical value of the proteolytic flux is usually larger than ~3, which corresponds to about 0.5 in log_10_ space.

**Figure 4:**
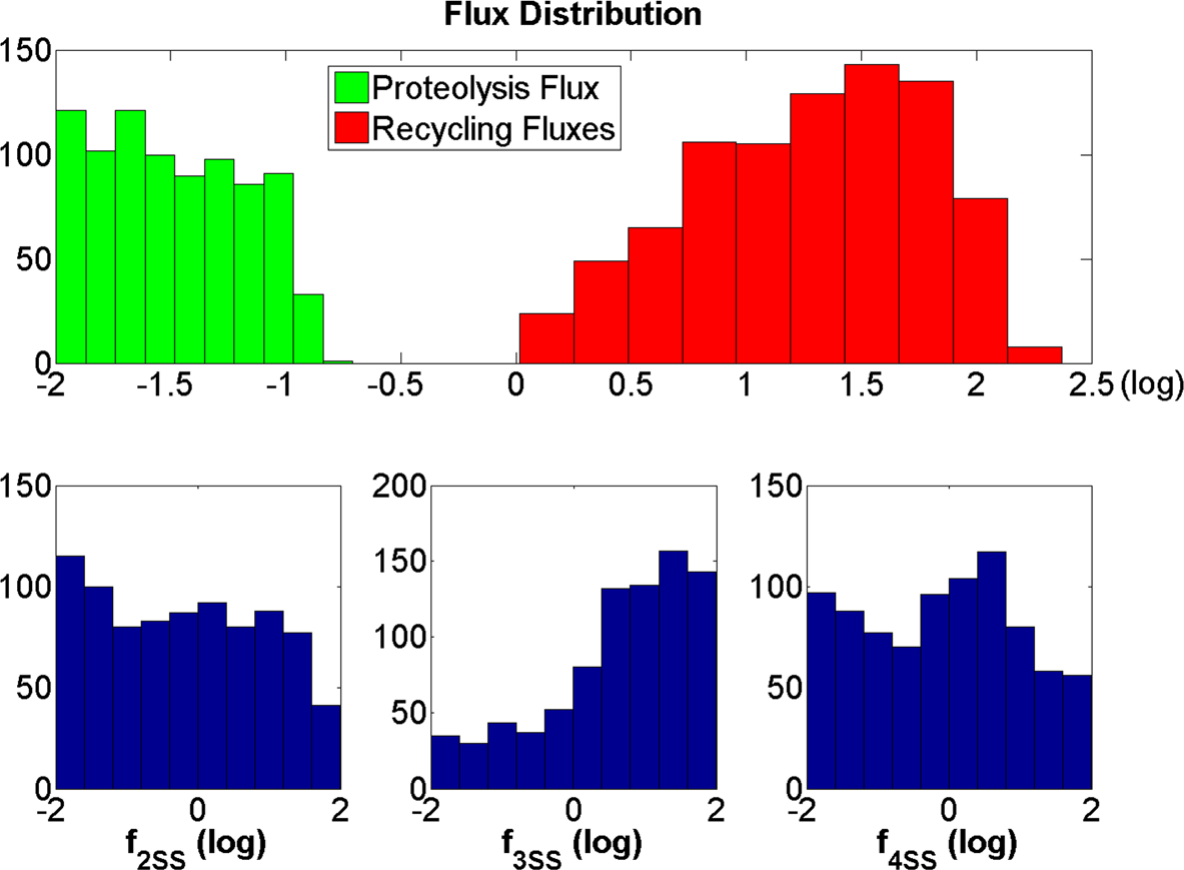
**Flux distributions for simulation samples satisfying three experimental observations.** Upper panel: flux distributions of proteolytic and recycling fluxes. Lower panel: distributions of magnitudes of individual recycling fluxes. The *x*-axis represents the different fluxes in log_10_ space, while the *y*-axis shows the observed count of sampled fluxes after filtering.

Valid combinations of flux magnitudes should satisfy all three experimental conditions. Thus, when we simultaneously screen against all three conditions, we obtain the result in the lower-right panel of Figure 3: the admissible flux combinations are compactly located in a well-defined region where the numerical value of the proteolytic flux is less than ~0.3 (corresponding to about −0.5 in log_10_ space) while the recycling flux is larger than 1. An extended flux distribution analysis (discussed below; see Figure 4) of these remaining points confirms this result.

To summarize this section, the fluxes were originally sampled from the same probability distribution. Filtering according to the three experiment-based conditions discussed before yields proteolytic and recycling fluxes that have distinctly separated distributions, with the average recycling flux exceeding the average proteolytic flux by almost 3 magnitudes in log space, which corresponds to a ratio of about 500 fold. In other words, the combined criteria-driven identification of suitable parameter ranges clearly suggests that under resting conditions (unstimulated cells) the dissociation of Nox1_active_ into recycled subunits plays a dominant role in lowering enzyme activity, thus serving as the primary disassembly mechanism. Beyond this conclusion, the flux distribution analysis of the recycling pathways *f*_2*SS*_, *f*_3*SS*_, and *f*_4*SS*_ so far does not reveal a clear activity pattern (lower panels in Figure 4).

### Patterns of Nox1 recycling mechanisms

As suggested by the results in previous sections, the three recycling pathways appear to play much more important roles for the disassembly of Nox1_active_ than proteolysis. In fact, the fluxes going through these pathways are at least 2 magnitudes (~100 fold) larger, a conclusion that is consistent no matter which of the discussed criteria are used. It is now of interest to explore whether any of the recycling fluxes is more effective than the others. For this analysis, we temporarily ignore the small contribution of proteolysis and set its flux *f*_14SS_ to zero. To balance this missing efflux from the system, the alleged input influxes *f*_11SS_, *f*_12SS_, and *f*_13SS_ are also set to zero, so that the constraints in Equation (4) of the *Methods* section are again satisfied.

**Figure 5:**
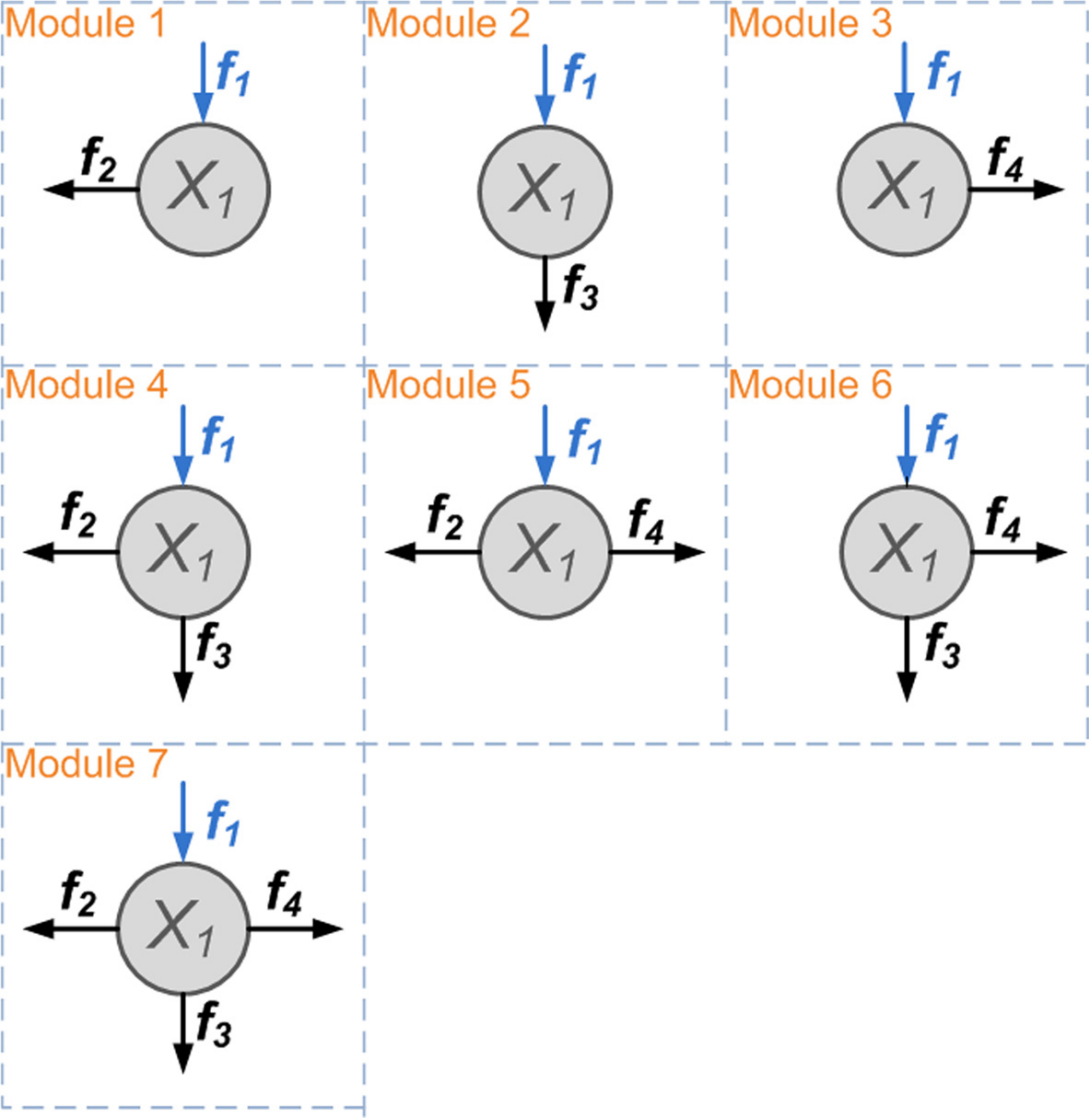
**Schematic representation of seven potential Nox1 recycling modules.** The proteolytic flux *f*_14SS_ is assumed to be negligible, as suggested by simulation results in previous sections.

Accounting for all combinations, seven design modules for the three recycling pathways of Nox1_active_ are possible (Figure 5). The steady-state fluxes through these pathways are *f*_2SS_, *f*_3SS_, and *f*_4SS_. *A priori* we do not have any information regarding the relative importance of each of these fluxes or their combinations, and we again use Monte-Carlo simulations reflecting treatment with AngII (represented by a combined *S*_1_ and *S*_2_ signal). The identification of superior or inferior module designs is achieved by combined comparisons of their responsiveness to input signals, as well as the response times of their “on” and “off” processes. Here, the response time is defined as the time interval within which the system numerically reaches a new steady state after a signal is applied, while “on” and “off” processes, respectively, refer to the responses after a signal is added or removed from the system.

We begin with a closed Nox1 system (for the model structure see Additional file 1: Figure S4), where the efflux *f*_16SS_ has such a small magnitude that it can be omitted. We analyze the system responses to a combined *S*_1_ and *S*_2_ input signal and also track how the active Nox1 complex behaves when the input signal is removed.

In order to keep the initial flux distribution consistent with observations in AngII treatment experiments, we set the control condition such that free p47^phox^, GDP-bound Rac1, and NoxA1 are dominant; in earlier notation, this setting corresponds to index 1 in Table 2. For fair comparisons among the different flux modules, we always set the flux of active Nox1 production (*f*_1SS_) constant (at a value of 100), which ensures that differences in system performance are due to the module structure at hand, rather than differences in flux magnitudes in a given Monte-Carlo sample. Within this constraint, the remaining independent fluxes (*f*_6*SS*_, *f*_8*SS*_, and *f*_9SS_) are calculated from (uniformly) randomly sampled rate constants γ_6_, γ_8_, and γ_9_. Sampling rate constants rather than fluxes allows us to inspect the effects of different reaction rates, especially those associated with reversible reactions.

**Figure 6:**
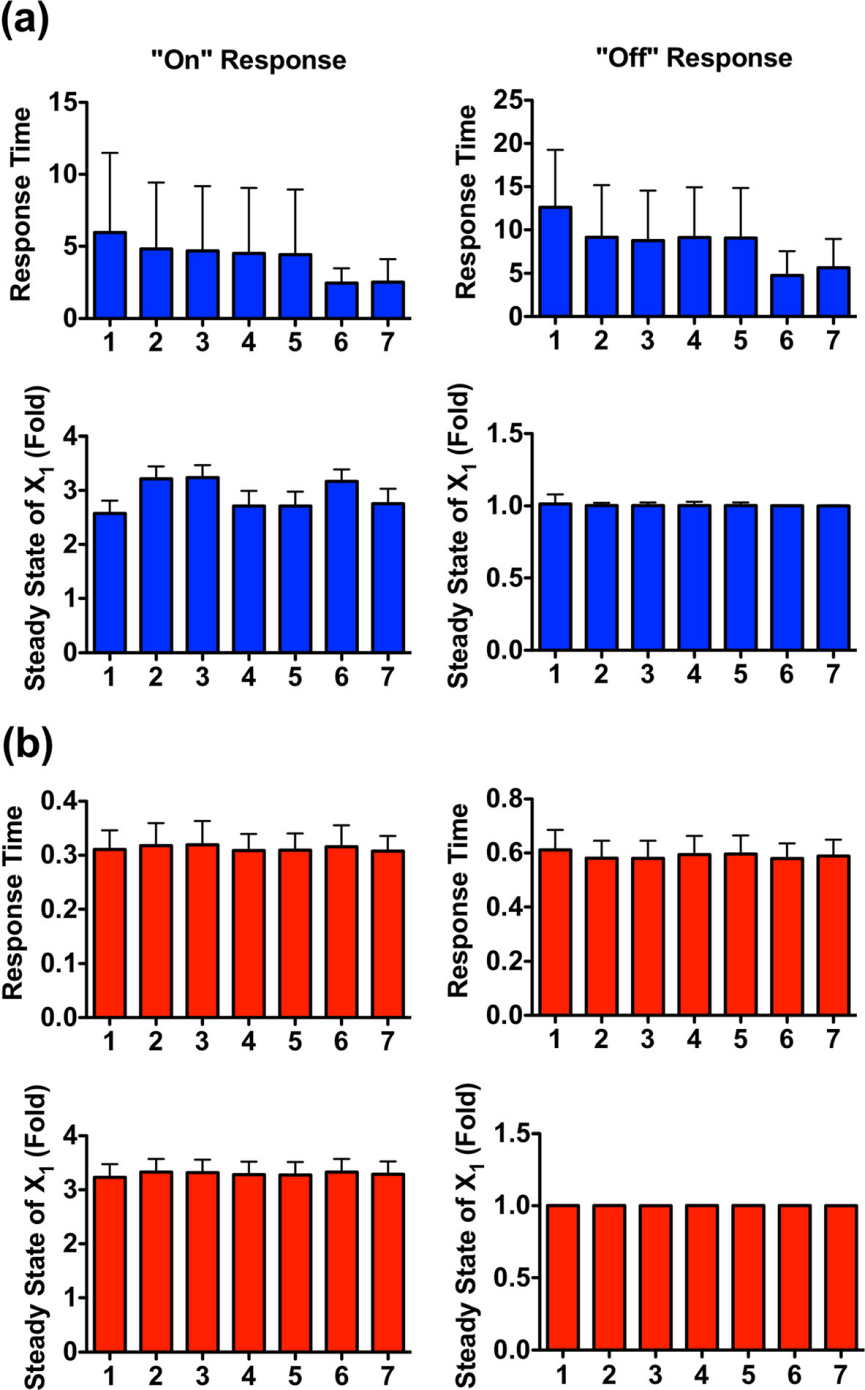
**Results of Monte-Carlo simulations of a closed Nox1 system (*f***_**16SS **_**= 0).** (**a**) Slow rates for reversible reactions with *γ*_*i*_ ∈ *U*(0, 1), *i* = 6, 8, 9. (**b**) Fast rates for reversible reactions with *γ*_*i*_ ∈ *U*(10, 100), *i* = 6, 8, 9. Integer numbers indicate the index of the recycling module. The sampling size for each bar is 2,000 points. Note different *y*-axes. Error bars are sample standard deviations.

The rate constants, which represent the reversible reactions for activation and deactivation of subunits p47^phox^, Rac1, and NoxA1, respectively, are either sampled as “slow” from the uniform distribution *U*(0, 1), or as “fast” from the uniform distribution *U*(10, 100). The simulation results (Figure 6a) indicate that for “slow” reversible reaction rates all seven disassembly modules tend to exhibit similar responsiveness. Namely, the steady state value of *X*_1_ in the “on” response is up-regulated to about the same level (between 2.5 ~ 3 fold) in all simulations, and returns to the control level (at “1”) when the input signal is removed in the “off” response.

Interestingly, the response times for the “on” and “off” responses are different among the modules. Module 1, which has only one recycling pathway, specifically associated with *f*_2SS_, has the longest response time in both the “on” and “off” responses. By contrast, modules 6 and 7, which contain both recycling pathways *f*_3SS_ and *f*_4SS_ have much shorter response times (less than half of other response times). The response time is an important feature of a signaling system, because it determines its refractory period. Thus, the latter modules are to be considered more efficient than the former.

When the reversible reaction rates within the pathway system are fast in both directions, the differences among all seven modules are rather small in terms of both responsivenss and response times (Figure 6b). Thus, these simulation results clearly demonstrate how relative reaction rates for the reversible processes in the system affect performance.

In reality, the system is open, and the efflux *f*_16SS_ may affect the disassembly of Nox1. The analysis of this case is complicated by the fact that we have no information regarding the relative magnitudes of the input fluxes and only know that the constraint *f*_2SS_ + *f*_9SS_ = *f*_10SS_ + *f*_16SS_ must be true. We study two scenarios. In the first, *f*_9SS_ is larger than *f*_10SS_ (which also means that *f*_2SS_ is smaller than *f*_16SS_), which tips the balance from NoxA1 to NoxA1_p_, while in the second scenario the opposite is true.

**Figure 7:**
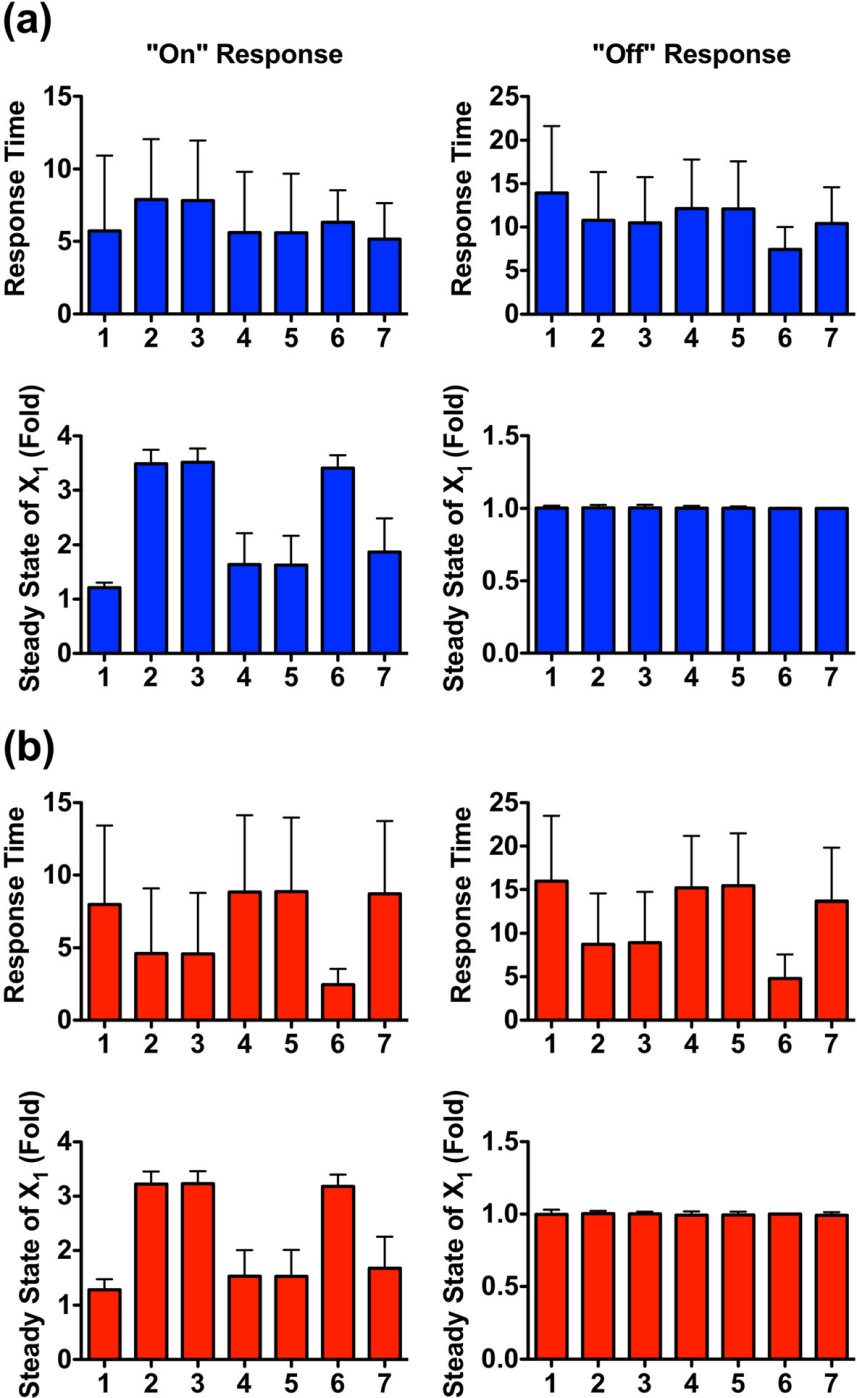
**Results of Monte-Carlo simulations for an open Nox1 systems with slow reversible reaction rates (*f***_**16SS **_**≠ 0).** (**a**) Scenario 1: *f*_9SS_ > *f*_10SS_; (**b**) Scenario 2: *f*_9SS_ ≤ *f*_10SS_. Reversible reaction rates *γ*_*i*_ ∈ *U*(0, 1), *i* = 6, 8, 9. Integer numbers indicate the index of the disassembly module. The sample size for each bar is 2,000 points. Error bars are sample standard deviations.

**Figure 8:**
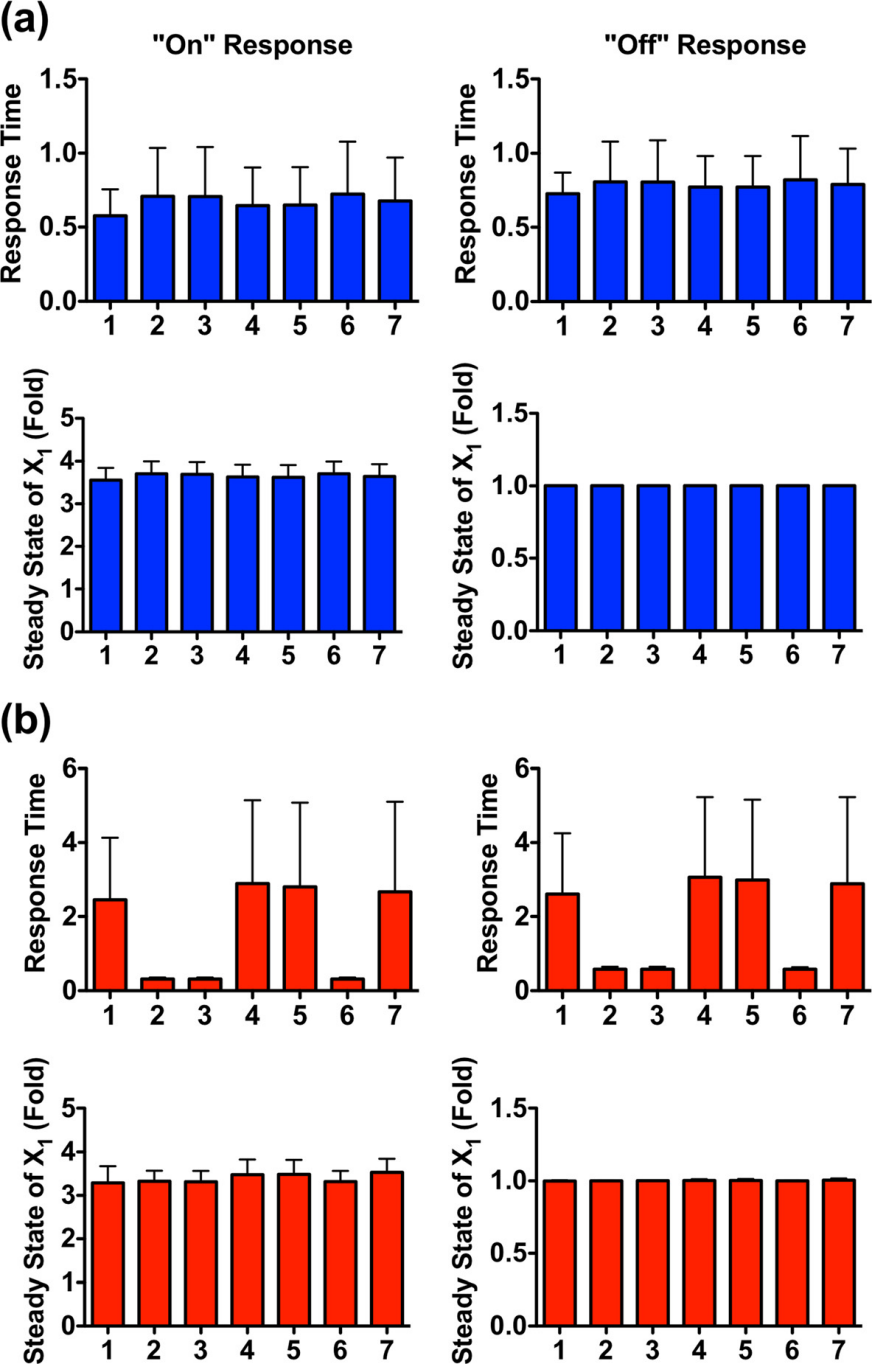
**Results of Monte-Carlo simulations for an open Nox1 system with fast reversible reaction rates (*f***_**16SS **_**≠ 0).** (**a**) Scenario 1: *f*_9SS_ > *f*_10SS_; (**b**) Scenario 2: *f*_9SS_ ≤ *f*_10SS_. Reversible reaction rates *γ*_*i*_ ∈ *U*(10, 100), *i* = 6, 8, 9. Integer numbers indicate the index of the recycling module. The sample size for each bar is 2,000 points. Error bars are sample standard deviations.

Intriguingly, the system performance is strongly affected by the transition from a closed to an open system (compare Figures 6a and 7 and Figures 6b and 8). In particular, the magnitudes of the reversible reaction rates now affect the performance of the system differently for the various disassembly modules. When the reversible reaction rates are slow (Figure 7a and b), different modules exhibit distinct responses. No matter the direction of the net flux between *f*_9SS_ and *f*_10SS_, for instance, modules 2, 3, and 6, which do not contain the recycling pathway *f*_2SS_, always have a higher up-regulated activity of *X*_1_ (with an averaged value above 3 fold) than modules 1, 4, 5, and 7 (with averaged values ranging from just above 1 to less than 2 fold).

In contrast, the direction of the net flux between *f*_9SS_ and *f*_10SS_ affects the patterns of response times among all disassembly modules. Good examples are again the stronger responders (modules 2, 3, and 6), which exhibit shorter response times (with the shortest average time at one third of the other reponse times) when *f*_9SS_ ≤ *f*_10SS_ (Figure 7b) but show insignificant differences in response times if *f*_9SS_ >*f*_10SS_ (Figure 7a).

When all reversible reaction rates are fast, the seven recycling modules tend to perform similarly in terms of responsiveness as well as response time (Figure 8a and b). However, the response times of modules 2, 3, and 6 are shorter than the others if *f*_9SS_ ≤ *f*_10SS_ (Figure 8b), indicating that this constraint renders these three modules particularly efficient.

## Discussion

Although Nox1 exhibits relatively low basal expression in VSMCs [28], it has been extensively studied. Nox1-induced ROS often serve as important intracellular signaling molecules, transducing extracellular signals into intracellular targets. This process can be activated by numerous physiological and pathological stimuli [27,28,49] and has therefore been implicated in a variety of cardiovascular diseases [24,33]. As a consequence, Nox1 must function properly and efficiently. It must be able to respond quickly and strongly. It is similarly important for Nox1 to return quickly to its unstimulated physiological state, which we called RtRS, lest it is unable to respond to further stimuli.

The activation and disassembly processes of Nox1 in VSMCs constitute a system of pathways whose components have been identified and characterized to some degree over the past decades [20,27,28,35,40,50]. In particular, the scientific community has assembled quite a detailed picture of how individual subunits of Nox1 are organized to form an active enzyme complex. While the components and basic interactions are known, the overall functionality of the Nox1 system is not easy to intuit. For instance, the balance between phosphorylated and dephosphorylated states under resting conditions, as well as the reaction rates of the system’s governing processes, might appear to be accidents of evolution: After all, why should it matter in which manner the active Nox1 complex is disassembled? As we have shown with our computational analysis here, specific routes of disassembly and proper characteristics of the unstimulated RtRS are crucial for optimal performance. To arrive at this insight, we set up a rather generic pathway model and analyzed it in a fashion that is only minimally affected by the choice of particular parameter values.

The model accounts for four potential Nox1 disassembly pathways, which include one direct degradation pathway of Nox1_active_ via proteolysis and three independent recovery pathways for subunits (see fluxes *f*_14_, *f*_2_, *f*_3_, and *f*_4_ in Figure 1). Investigations of these fluxes under resting conditions were performed with Monte-Carlo simulations. For these analyses, the relatively effectiveness of the four recovery pathways was assessed in terms of different criteria, namely, the strength and speed of system responses to input signals, as well as their consistency with experimental and clinical data.

Interestingly, assessments with respect to the different criteria led to similar results. First, for effective functioning, the flux associated with proteolysis (*f*_14SS_) must be much smaller than the fluxes associated with recycling pathways (Figures 2, 3, 4), indicating the relative inefficiency of proteolysis for the disassembly of Nox1_active_ under resting conditions. Expressed differently, the disassociation of the active Nox1 complex and the concomitant recycling into subunits play the dominant role, which is seen in the relatively large magnitudes of these fluxes. This result is not too surprising, because proteolysis requires total *de novo* assembly of Nox1 before new signals can be transduced.

Less predictable, the optimal RtRS is characterized by specific balances between phosphorylated and dephosphorylated forms of the subunits. These proper balances are critical for efficiency. As consistently shown by simulation results for different input signals (Figure 2a to d), the initial states with indices 1 and 2 in Table 2 exhibit the most effective responses to external signals, while systems with indices 7 and 8 in Table 2 are the least efficient. These results imply that both p47^phox^ and Rac1 should mainly reside in their inactive forms when the system is at rest. This inference from the computational analysis is well consistent with so-far unexplained experimental observations [41,47,48] and offers an unbiased rationale and explanation. As an interesting side effect, this state equips the Nox1 system with a certain degree of noise tolerance: Residing in the inactive forms, any spurious formation of Nox1 in the absence of true signals, followed by erroneous signal transduction, is much less likely than if the system maintains the active forms of subunits, which are the direct precursors of the active Nox1 enzyme. Thus, to avoid spurious signaling events, the system is “parked” in its inactive form and requires external stimuli to trigger a signal transduction event. In contrast to p47^phox^ and Rac1, the unphosphorylated form of the Nox1 activator unit NoxA1 is the direct precursor component of active Nox1, and the current analysis has not revealed whether the balance is tilted toward the precursor or its complement.

These computational findings are solid for the system analyzed here. They can also be seen as hypotheses that are to be tested in laboratory experiments, because it is of course possible that the model assumptions made here are not entirely correct or complete. Thus, one hypothesis is the minor role of the proteolytic flux, while other hypotheses result from the inferred relative magnitudes of three recycling pathways, the speed of the reversible reactions within the system, and the dominance of inactive states at rest.

One should note that although the three disassociation and recycling pathways apparently play interchangeable roles in disassembling Nox1_active_, they actually guide the system to different states. It is instructive to perform another thought experiment to study the resulting consequences. Suppose the system receives a strong signal *S*_1_. Depending on the different combinations or flux distribution patterns among the three recycling pathways *f*_2_, *f*_3_ and *f*_4_, the enzymatic profiles of Nox1_active_ generation can vary substantially. Let’s first consider the extreme case where only *f*_3_ is present as the recycling pathway (*cf.* Figure 1). In the presence of *S*_1_, p47^phox^ becomes depleted, and formation of active Nox1 subsequently depletes the other active precursors Rac1_GTP_ and NoxA1. Disassociation of the Nox1 complex through pathway *f*_3_ restores p47^phox^ and, more importantly, Rac1_GTP_ and NoxA1, which enable sustained formation of new active Nox1 as long as *S*_1_ is present. In other words, if *S*_1_ is persistent and *f*_3_ is the only recycling pathway, Nox1 will be highly and continuously activated. By contrast, if *S*_1_ is persistent and the system does not use *f*_3_, but instead *f*_2_ or *f*_4_, the situation is very different. Because *f*_2_ and *f*_4_ respectively restore NoxA1p and Rac1_GDP_, but not NoxA1 and Rac1_GTP_, the restoration of these two active precursors depends mainly on the reverse reactions *f*_7_ and *f*_10_. As a consequence, the formation of new active Nox1 is now limited by the availability of NoxA1 or Rac1_GTP_ even if there are significant residual amounts of phosphorylated p47^phox^. As a result, one should expect a much lower activity of Nox1, compared to the previous case, even under the same input signal *S*_1_. In other words, in the presence of all three pathways, the system can lower Nox1 activity by either inhibiting *f*_3_ or decreasing its flux distribution ratio in the presence of input signal *S*_1_.

The analogous arguments hold for an input signal of type *S*_2_. By contrast, signal *S*_3_ is a “negative” signal that effectively shuts down the entire system as it decreases the availability of NoxA1. The presence of pathway *f*_2_ can further exacerbate this situation, while the presence of *f*_3_ or *f*_4_ may counteract this negative effect by releasing more active NoxA1. However, the situation under this scenario is more complicated because *f*_3_ or *f*_4_ also restore the inactive form of p47^phox^ or Rac1_GDP_, which might need additional activation signals. These insights from thought experiments can be considered specific hypotheses that are to be validated or refuted experimentally.

Nothing is truly known about the internal regulation of the Nox1 system, but our analysis implies that the three disassembly and recycling pathways may not just be redundant variants, but that they may permit specific control mechanisms that allow the system to habituate, adjust to persistent signals, and control its refractory period under repeated signals of the same or different types. As an example, we discussed a system exposed to a persistent signal *S*_1_ and comparative simulation results for three distinct scenarios with only one recycling pathway each (for further details, see text and Additional file 1: Figure S1). Although the responsiveness of the Nox1 system is affected by a number of factors, such as the relative rates of the reversible reactions and the magnitude of efflux, recycling via dephosphorylation of p47^phox^ consistently tends to lead to as high, and usually higher, a steady state of *X*_1_ than the other two recycling mechanisms. Therefore, to achieve enhanced responsiveness to persistent signals of type *S*_1_, the most efficient regulatory mechanism would appear to be the activation of the corresponding recycling pathway via dephosphorylation of p47^phox^. This activation could be triggered directly by signals of type *S*_1_ or by an indirect mechanism. Particular responsiveness to other signals would require different control strategies.

In order to explore the relative effectiveness of the three recycling pathways, we used Monte-Carlo simulations to test the performance of seven distinct disassembly modules. These modules accounted for scenarios with only one, two or all three recycling pathways. Initially, we tested the case where the basal flux through the proteolytic pathway (*f*_14_) is so small that it can be omitted. Several scenarios were explored and three intracellular settings, including the speed of reversible reactions, the magnitude of efflux *f*_16_, and the net balance between the fluxes converting NoxA1 and NoxA1_P_ into each other, were investigated. A comprehensive comparison of all simulation results revealed that the first factor, the speed of the reversible reactions, most strongly affects the relative performance of all seven disassembly modules. When the reversible reaction rates are very fast, all modules tend to exhibit a similar performance in terms of responsiveness and response times for almost all tested scenarios (Figures 6b and 8).

There is one important exception. When the efflux *f*_16_ is not zero and the flux *f*_9_ is smaller than *f*_10_ (in other words, if *f*_2_ ≥ *f*_16_ > 0), modules 2, 3, and 6 perform better, with shorter response times than others modules. This result strongly implies that in order to achieve an effective response, it is crucial to replenish the subunit pools as quickly as possible. Due to the fast reversible reaction rates, each individual subunit can rapidly be recycled and becomes available again. As a consequence, it becomes less important in which way Nox1_active_ disassociates and the pathway recycles its subunits.

By contrast, when the reversible reaction rates are relatively slow, the relative performance of the disassembly modules varies from one scenario to another, and the question of which design is optimal depends on a combination of the magnitude of efflux *f*_16_ and the net balance between the fluxes converting NoxA1 and NoxA1_P_ into each other. As shown in Figure 7a and b, modules 2, 3, and 6 exhibit better performance (~3 times more responsive than other designs) when the efflux *f*_16_ cannot be ignored. The superiority of these three modules is strenghtened by their shorter response times, especially when the flux *f*_9_ is smaller than *f*_10_ (Figure 7b). However, if the efflux *f*_16_ is small enough to be ignorable (Figure 6a), all seven modules tend to have similar performances in terms of the system’s responsiveness. In this case, the optimality of different design modules is only determined by the response times, and modules 6 and 7 can be identified as the superior designs (Figure 6a).

These results and interpretations clearly show the strong relationships between the intracellular process organization and the overall functionality of the system. Clearly, tipping the balance toward the phosphorylated form supplies substrate for *f*_16_, thereby leading to a depletion of this subunit. This effect is exacerbated by a large apportionment of flux toward *f*_2_, which is the only recycling flux generating NoxA1_P_. Indeed, supporting this assessment, our model analysis points to the importance of a suitable balance between *f*_9_ and *f*_10_. Given the potentially detrimental outcome associated with this scenario, it will be useful to study the magnitudes and regulation of both *f*_16_ and *f*_2_ experimentally.

## Conclusions

We have employed computational methods to investigate the effectiveness of alternative strategies for the disassembly of Nox1 and the recycling of its subunits in VSMCs. The results of the Monte-Carlo simulations, which by and large are independent of specific parameter values, strongly indicate that disassembly through recycling is more effective than proteolysis. Furthermore, the relative performance of each recycling pathway is affected by the rates and balances of the reversible reactions in the system, as well as the magnitude of the efflux of phosphorylated NoxA1 (*f*_16_) and the direction of the net flux between NoxA1 and NoxA1_P_. These balances are natural targets for internal control mechanisms of Nox1 dynamics, which at this point are unknown. The computational inferences directly form targeted hypotheses that can and should be tested with laboratory experiments.

## Methods

### Features of Nox1 in VSMCs

NADPH oxidase 1 (Nox1) is expressed at the plasma membrane [25,51] and in endosomes [52] of vascular smooth muscle cells (VSMCs) of large vessels. For protein stabilization and enzymatic activity, Nox1 needs to associate with the membrane subunit p22^phox^[50,53], thereby forming a transmembrane complex. Nox1 is activated when it is complexed with the cytosolic regulators p47^phox^[33,36] and NoxA1 [39,40], and the small GTPase Rac1 [35,54,55]; the process is denoted with *f*_1_ in Figure 1. Activation of Nox1 is usually preceded by the activation of its cytosolic regulators. Activation of p47^phox^ and Rac1 by certain upstream signals is essential for obtaining full enzymatic activity of Nox1 in VSMCs. In unstimulated cells, p47^phox^ mainly resides in the cytosol [41,47]. It requires additional phosphorylation in several positions to open its auto-inhibitory region and to turn it into its active form [20,56], which then enables it to translocate from the cytosol to the membrane where it associates with the Nox1/p22^phox^ complex.

The translocation of phosphorylated p47^phox^ often occurs together with the activator subunit NoxA1 [20,40]. This phosphorylation of p47^phox^ is mainly catalyzed by protein kinase C (PKC) [27,57] (marked *f*_5_ in Figure 1), which may be stimulated by AngII, typically in association with three different phospholipases [46,58,59]. However, there are exceptions. For instance, the strong PKC activator PMA alone was shown to increase Nox1 activity in VSMCs [16]. Similar to the activation of p47^phox^, the small GTPase Rac1 also requires activation by certain upstream signals. Also like p47^phox^, Rac1 can exist in two interconvertible forms: GTP-bound (active) and GDP-bound (inactive). Activation of Rac1from its inactive form is facilitated by a family of enzymes called Guanine Exchange Factors (GEFs) [48] (associated with *f*_7_ in Figure 1). GEFs render Rac1 capable of associating with the Nox1/p22^phox^ complex, which is another indispensable step for achieving enzymatic activity in Nox1 [35]. Interestingly, the action of GEFs on Rac1 is usually independent of the translocation of p47^phox^[48]. As an alternative, recent studies suggest that the second cytosolic subunit, NoxA1, can be phosphorylated by protein kinase A (PKA) [44,60] (process *f*_9_ in Figure 1). The phosphorylation of NoxA1 greatly enhances its binding affinity to 14-3-3 proteins, thus preventing its association with other Nox1 subunits. Although this mechanism has not directly been verified in VSMCs, it has been suggested as a possible negative regulatory mechanism of Nox1 since both the signal (PKA) and the substrate (NoxA1) co-exist in this type of cell.

Many experimental studies have investigated Nox1-induced ROS production in different cells and under various conditions. By comparing the system responses to certain stimuli with the control system, several Nox1 activators and regulators have been identified. For instance, Nox1 can be directly activated by PDGF [28], AngII [27,28], thrombin [20], tumor necrosis factor-alpha **(**TNF-α) [52], and many others (for details see [24,33]). In these experiments, the measured output variables are usually intra- or extra-cellular ROS concentrations or production rates under different conditions. For example, AngII (100 nmol/L) has been shown to increase Nox1-catalyzed ROS significantly [27,46], and it is therefore considered an effective activator of Nox1 enzyme. However, due to differences in experimental settings, the measured ROS concentration or production might be found in different magnitudes, and it is possible to encounter slightly different transient behaviors even in response to the same stimulus. As an example, two experimental groups treated cells with AngII and measured the response over a period of 30 minutes. In both cases, the ROS concentration eventually reached a higher plateau (~2 fold), but in one case the transient was found to be biphasic [27], while the other group indicated a continuous and gradual increase in ROS concentration to the same plateau [46]. In both cases, the plateau was sustained for a long time indicating that the levels of ROS and of the corresponding active Nox1 complex assume an up-regulated steady state in response to AngII treatment that is higher than in controls. Western blot analysis did not show any significant effect of AngII on the total Nox1 protein level over 4 hours after AngII treatment [26], which implies that ROS production is likely due to AngII-induced signal activation of Nox1 activity rather than to an increase in the Nox1 protein level.

### Variables, assumptions, simplifications, and model settings

The Nox1 activation system has been investigated in numerous studies, and the pathway structure, key components, and a variety of other biological data are available in the literature. The model we propose is based on this information and directly reflects the pathway structure shown in Figure 1. The most pertinent information about the model is summarized in Table 3. The dependent variables, whose concentrations are affected by the system, include active Nox1 complex (Nox1_active_), Nox1/p22^phox^, p47^phox^, phosphorylated p47^phox^ (p47^phox^_P_), GDP-bound Rac1 (Rac1_GDP_), GTP-bound Rac1 (Rac1_GTP_), NoxA1, and phosphorylated NoxA1 (NoxA1_P_). Input signals, including those leading to p47^phox^ phosphorylation, those effecting Rac1 activation, and those responsible for NoxA1 phosphorylation, are represented by *S*_1_, *S*_2_, and *S*_3_, respectively. They are treated as independent variables, which nominally stay at 1 for control conditions and are changed to a value above 1 when a relevant extracellular stimulus is applied.

**Table 3:** System variables

**Variable**	**Biological component**
*X*_*1*_	active Nox1 complex (Nox1_active_)
*X*_*2*_	Nox1/p22^phox^
*X*_*3*_	p47^phox^
*X*_*4*_	phosphorylated p47^phox^ (p47^phox^_P_)
*X*_*5*_	GDP-bound Rac1 (Rac1_GDP_)
*X*_*6*_	GTP-bound Rac1 (Rac1_GTP_)
*X*_*7*_	NoxA1
*X*_*8*_	phosphorylated NoxA1 (NoxA1_P_)
*S*_1_^*^	signals that phosphorylate p47^phox^, such as protein kinase C (PKC)
*S*_2_^*^	signals that activate Rac1
*S*_3_^*^	signals that phosphorylate NoxA1, such as protein kinase A (PKA)

^*^ indicates independent variables; others are dependent variables.

In a VSMC system residing in its control state and exposed to basal input signals, one detects a continuous, low production of ROS [20,26]. This ROS production does not decrease much even if Nox4, a Nox homologue, is significantly knocked down [26]. Combining these two observations suggests the sustained existence of Nox1_active_ even if it might have a low concentration. It also indicates that the target system at the ready-to-respond state (RtRS) dynamically maintains a fine-tuned balance of fluxes. As an illustration, consider Nox1_active_ (*X*_1_). Basal signals enable continuous formation of *X*_1_ by depleting the pool of Nox1/p22^phox^, p47^phox^_P_ , Rac1_GTP_, and NoxA1_P_. This formation continues to exist as long as the basal signals are present and the pool of free subunits is not depleted. Thus, in order to maintain *X*_1_ at a stable level, there must be additional pathways (generically referred to as disassembly pathways) that disassociate *X*_1_.

Four disassembly pathways are proposed in the current model. They include one direct degradation pathway via proteolysis (associated with *f*_14_ in Figure 1) and three independent recycling pathways that recoup subunits of Nox1 (associated with *f*_2_, *f*_3_, and *f*_4_ in Figure 1). Because the exact enzymes that catalyze these disassembly reactions of Nox1_active_ have not been clearly identified in VSMCs, and their responses to extracellular stimuli are ill characterized, these enzymes are modeled with a nominal activity level of 1. The same strategy is applied to partial or more complicated disassociation mechanisms of Nox1_active_, which might exist to serve as effective disassembly mechanisms.

Initial ranges for the Monte-Carlo simulations could not always be obtained from experimental data. In the absence of experimental values, we were forced to use more or less arbitrary default values. Sensitivity analysis (see details in Additional file 1: Section 6) shows that these values do not qualitatively affect later results and comparisons of scenarios. In particular, these initial ranges typically do not affect the steady state, which is of interest in the later analyses, although they do affect the speed with which the steady state is reached.

As an example, the initial upper boundary for fluxes was set to 100, but the analysis showed that we could have chosen 10 or 1000 instead, without affecting the later filtering results. Thus, we started with generous, but reasonable ranges and gradually decreased them where feasible.

In terms of signal strengths, any signal with a value above 1 is considered a stimulus to the system. As far as experimental information permitted, we used realistic values as input signals (for instance, see Additional file 1: Section 3 for settings of input signals in AngII and PMA treatments). If no specific experimental data were available, we used an input signal intensity of 10, in order to differentiate the comparative result most clearly.

### Model structure

Based on the model assumptions and the simplifications described above, we propose a model structure, which directly corresponds to the scheme in Figure 1, to represent the dynamics of Nox1 in VSMCs. In order to balance the loss of material leaving the system via the proteolytic pathway of Nox1_active_, the model accounts for several processes entering the system, including influxes of Nox1/p22^phox^, p47^phox^, Rac1_GDP_, and NoxA1 (blue arrows marked *f*_11_, *f*_12_, *f*_13_, and *f*_15_ in Figure 1, respectively). Because the PKA-phosphorylated NoxA1 (NoxA1_P_) has been shown to bind with 14-3-3 protein to form a complex [60], which could be directly degraded, one additional degradation pathway of NoxA1_P_ (associated with *f*_16_ in Figure 1) is added to the system. All reactions between active and inactive forms of individual subunits (marked *f*_6_, *f*_8_, and *f*_10_ in Figure 1), such as dephosphorylation of p47^phox^ and NoxA1, association of GDP to inactivate Rac1, are considered to be reversible, which is supported by experimental observations [20,44,48]. By accounting for these various influxes and effluxes, the pathway constitutes an open system that operates at a dynamic steady state, with small amounts of material continuously flowing in and out of the system.

### Mathematical description

The first and arguably most critical step of biological model design is the translation of the system diagram into an appropriate mathematical structure that permits analytical or simulation-supported diagnosis [61]. We use here ordinary differential equations in mass action format that describe how each component in the system varies over time.

The Nox1 system represented in Figure 1 can be expressed succinctly in matrix format:

(2) $$\dot{X}=N\cdot F$$

where *X*_8×1_ is the dependent variable vector, $\dot{X}$ denotes its derivative, *F*_16×1_ is the flux vector, and *N*_8×16_ is the stoichiometry matrix, which quantifies the relationships between dependent variables and corresponding fluxes (see details of *N* in Additional file 1). The fluxes are given in mass action format as

(3) $$\begin{array}{llll} f_{1}=\gamma_{1}X_{2}X_{4}X_{6}X_{7} & f_{5}=\gamma_{5}X_{3}S_{1} & f_{9}=\gamma_{9}X_{7}S_{3} & f_{13}=\gamma_{13}\\ f_{2}=\gamma_{2}X_{1} & f_{6}=\gamma_{6}X_{4} & f_{10}=\gamma_{10}X_{8} & f_{14}=\gamma_{14}X_{1}\\ f_{3}=\gamma_{3}X_{1} & f_{7}=\gamma_{7}X_{5}S_{2} & f_{11}=\gamma_{11} & f_{15}=\gamma_{15}\\ f_{4}=\gamma_{4}X_{1} & f_{8}=\gamma_{8}X_{6} & f_{12}=\gamma_{12} & f_{16}=\gamma_{16}X_{8} \end{array}$$

### Parameter estimation

Model simulations and system diagnosis require the specification of numerical values for all parameters from experimental observations. This step is often challenging, for a wide variety of reasons (*e.g.*, see [62]). In the present case, the Nox1 system is at first assumed to operate at its nominal, stable steady state (RtRS), which corresponds to unstimulated control conditions. The concentrations of all dependent variables remain unchanged in this state, which allows the formulation of linear constraints that the fluxes must satisfy Equation (4).

(4) $$\left\{ \begin{array}{l} f_{1\mathrm{SS}}=f_{2\mathrm{SS}}+f_{3\mathrm{SS}}+f_{4\mathrm{SS}}+f_{14\mathrm{SS}}\\ f_{5\mathrm{SS}}=f_{3\mathrm{SS}}+f_{6\mathrm{SS}}+f_{11\mathrm{SS}}\\ f_{7\mathrm{SS}}=f_{4\mathrm{SS}}+f_{8\mathrm{SS}}+f_{12\mathrm{SS}}\\ f_{9\mathrm{SS}}+f_{2\mathrm{SS}}=f_{10\mathrm{SS}}+f_{16\mathrm{SS}}\\ f_{11\mathrm{SS}}=f_{14\mathrm{SS}}\\ f_{12\mathrm{SS}}=f_{14\mathrm{SS}}\\ f_{13\mathrm{SS}}=f_{14\mathrm{SS}}\\ f_{15\mathrm{SS}}=f_{14\mathrm{SS}}+f_{16\mathrm{SS}} \end{array}\right.$$

This set of constraints has an eight-dimensional basis, indicating that only eight linearly independent fluxes need to be characterized from data instead of sixteen. In addition, the fluxes are naturally non-negative, which imposes a set of inequality constraints that bound the search space for feasible parameter values further. Note that flux *f*_14*SS*_ disassembles NoxA1_active_ into all four subunits, which introduces a stoichiometric factor of 4 into the system, which becomes evident if the last four equations in (4) are summed.

Linear algebra assures us that it ultimately does not matter which basis of flux vectors in Equation (4) is chosen. For reasons of practicality, we select fluxes *f*_2*SS*_, *f*_3*SS*_, *f*_4*SS*_, *f*_6*SS*_, *f*_8*SS*_, *f*_14*SS*_, and *f*_16*SS*_, as independent fluxes. As the eighth independent flux, we choose *f*_9*SS*_ or *f*_10SS_, depending on the relative sizes of *f*_2SS_ and *f*_16SS_. Two reasons support this particular selection. First, *f*_2*SS*_, *f*_3*SS*_, *f*_4*SS*_, and *f*_14*SS*_ each represent one disassembly mechanism of Nox1_active_; these fluxes are therefore of particular interest. Their characterization will enable us to identify the most effective strategies of degrading Nox1_active_. Second, once these selected fluxes are numerically quantified by their rate constants, the remaining fluxes are guaranteed to be non-negative because of their relatively larger magnitudes.

Since little experimental information is available regarding any of the fluxes or their corresponding rate constants, we use Monte-Carlo methods, which permit the exploration of large search spaces with reasonable effort. Specifically, the targeted fluxes are randomly sampled from a uniform distribution over an appropriate non-negative domain. The performance of each sampled set is then evaluated by a series of user-defined criteria.

## Abbreviations

(Nox1): NADPH oxidase 1; (ROS): Reactive oxygen species; (eNOS): Endothelial nitric oxide synthase; (AngII): Angiotensin II; (VSMCs): Vascular smooth muscle cells; (PDGF): Platelet derived growth factor; (RtRS): Ready-to-Respond State; (PMA): Phorbol-12-myristate-13-acetate; (PKC): Protein kinase C; (GEFs): Guanine exchange factors; (PKA): Protein kinase A; (TNF-α): Tumor necrosis factor-alpha.

## Competing interests

The authors declare that they have no competing interests.

## Authors’ contributions

WY and EOV developed the concepts for analyzing the design of the pathway as a driver of effective functioning. WY implemented the models and performed the simulations and analysis. WY and EOV wrote the manuscript. Both authors read and approved the final manuscript.

## Supplementary Material

Additional file 1 The file contains supplementary information regarding the stoichiometric matrix of the system, simulation settings, and a detailed parameter sensitivity analysis, as well as Figures S1-S13,Tables S1 and S2. Click here for file
