# Supplementary material for: Function and design of the Nox1 system in vascular smooth muscle cells
Source: BMC Syst Biol. 2013 Mar 11;7:20. doi: 10.1186/1752-0509-7-20 (PMC3606394; doi:10.1186/1752-0509-7-20)
Supplement: Additional file 1 — The file contains supplementary information regarding the stoichiometric matrix of the system, simulation settings, and a detailed parameter sensitivity analysis, as well as Figures S1-S13,Tables S1 and S2. [file 1752-0509-7-20-S1.docx]

**Additional File 1**

**Function and Design of the Nox1 System in Vascular Smooth Muscle Cells**

**Weiwei Yin and Eberhard O. Voit**

***1. Stoichiometric Matrix***

The matrix *N* in Equation (2) is explicitly given as follows:

***2. Input signal vectors for the assessment of Nox1 disassembly (first criterion)***

The input signal vector ([*S*_1_ *S*_2_ *S*_3_]) is represented by the numerical values listed in Table S1.

**Table S1. Numerical values of input signals under different scenarios**

| Experimental  Condition | *S*_1_ signal  active | *S*_2_ signal  active | *S*_3_ signal  active | *S*_1_& *S*_2_ signals  active |
| --- | --- | --- | --- | --- |
| Control | [1 1 1] | [1 1 1] | [1 1 1] | [1 1 1] |
| Stimulated | [10 1 1] | [1 10 1] | [1 1 10] | [10 10 1] |

***3. Assessment of Nox1 disassembly mechanisms according to the second criterion***

Numerical values of initial conditions were assigned according to experimental observations (in consultation with experimentalists); they are summarized in Table S2. The experimental treatment with angiotensin II (AngII) is translated into a combination of input signals *S*_1_ and *S*_2_, while the treatment with 12-myristate-13-acetate (PMA) is translated into input signal *S*_1_. Considering realistic magnitudes with which AngII and PMA up-regulate these signals, the numerical values of *S*_1_ and *S*_2_ are conservatively considered as ~ 2 fold over control level (as “1”) for AngII, and the numerical value of *S*_1_ is considered as ~4 fold over control level for PMA. Therefore, the input signals of AngII and PMA treatments are respectively represented by [2 2 1] and [4 1 1] in this set of simulations.

**Table S2.**

**Unstimulated Steady-state values dependent variables; used as initial conditions**

| Variable | Steady-State Value |
| --- | --- |
|  | (molecules/cell)  |
|  | (molecules/cell)  |
|  | (molecules/cell)  |
|  | (molecules/cell)  |
|  | (molecules/cell)  |
|  | (molecules/cell)  |
|  | (molecules/cell)  |
|  | (molecules/cell)  |

The values in Table S2 are not directly available from the literature and therefore somewhat uncertain. In spite of many years of substantial experimental work, it is still only possible to measure the relative abundances of these variables, but not their absolute values. After extensive discussions with experts in the field, we decided to set the initial values based on the following semi-quantitative information. The amount of active enzyme (*X*_1_) is very low (about at, or just below, the detection limit). The quantities of subunits p47^phox^ (including both phosphorylated (*X*_4_) and unphosphorylated (*X*_3_) forms), Rac1 (including both active (*X*_6_) and inactive (*X*_5_) forms), and NoxA1 (including both free (*X*_7_) and phosphorylated (*X*_8_) forms) are more abundant. The transmembrane component *X*_2_ is believed to be the most abundant. Based on this semi-quantitative information, the quantity of *X*_1_ was assumed to be low, and represented with a nominal level of 1,000 units. The total of each type of convertible subunits (p47^phox^, Rac1 and NoxA1) was initially set several times higher. Specifically, the total of subunit NoxA1 (*X*_7_ + *X*_8_) was assumed to be 6,000, the total of subunit p47^phox^ (*X*_3_+*X*_4_) was set 10% higher, at 6,600, and the quantity of Rac1 (*X*_5_+*X*_6_) was initiated even higher, at 7,200. Furthermore, the two forms of each convertible subunit (active or inactive) are not evenly distributed. The experimentally observed dominant forms are unphosphorylated p47^phox^ (*X*_3_), Rac1_GDP_ (*X*_5_), and NoxA1 (*X*_7_). Therefore, the initial quantities of *X*_3_, *X*_5_, and *X*_7_ were set as 5,400 (~80%), 6,000 (~80%), and 5,400 (~90%), respectively. Correspondingly, the initial quantities of *X*_4_, *X*_6_, and *X*_8_ were set as 1,200 (~20%), 1,200 (~20%), 600 (~10%). Our experimental collaborators found these relative settings reasonable. Finally, the quantity of *X*_2_ was assumed to be 20 fold higher than *X*_1_.

While these initial settings contain a higher degree of uncertainty than one may like, sensitivity analysis showed that the precise values do not significantly affect the simulation results and conclusions.

The second criterion consists of a combination of three experimental conditions. They are separately implemented in simulations as defined below.

1. The first criterion, namely Nox1 protein conservation after 30 minutes of AngII treatment, is implemented as

.

1. The second criterion of a (quasi-) steady state and up-regulated ***X*_1_** after 30 minutes of AngII treatment is implemented as:

(i)

(ii) .

1. The third criterion of up-regulated *X*_1_ under PMA treatment is implemented as

.

***4. A thought experiment illustrating responses of the Nox1 system to persistent signals of type S_1_ under three extreme scenarios***

In order to illustrate the distinct roles of the three disassembly and recycling pathways in affecting the responses of the Nox1 system, we perform the following thought experiment. Suppose the Nox1 system is exposed to a persistent signal *S*_1_, and only one recycling pathway is available, as depicted with modules 1, 2 and 3 in Figure 5 of the Text. Specifically, in scenario 1, only the recycling pathway via phosphorylation of NoxA1 is available (marked as *f*_2_ in Figure1 of the Text). In scenario 2, only the recycling pathway via dephosphorylation of p47^phox^ (marked as *f*_3_ in Figure1) exists. In scenario 3, only the recycling pathway via deactivation of Rac1_GTP_ (marked as *f*_4_ in Figure1) is present. The system’s responses to *S*_1_ are evaluated with respect to the new steady state of *X*_1_ in one scenario compared to that in another scenario.

In all cases, the simulation settings are the same in terms of other parameter values and initial conditions for each round of simulation. Moreover, to reduce other possible discrepancies induced by different simulation settings, parameters and initial conditions are generated through uniform sampling within a relatively large range. For different rounds of simulations, parameters are randomly sampled as follows: The independent rate constants *γ*_i_ are sampled from *U*(0, 100), for *i* = 6, 8, 10, 16. Under different scenarios, *γ*_2_ (or *γ*_3_ or *γ*_4_) is respectively set at 10. The steady states of dependent variables in unstimulated state are set as: *X*_1_ = 10, *X*_2_ = 100, *X_i_* ~ *U*(1, 99) for *i* = 3, 4, …8 with the assumption that *X*_3_ and *X*_4_, *X*_5_ and *X*_6_, *X*_7_ and *X*_8_, respectively, sum to 100. The overall sampling size is 10,000. All simulations were implemented in MATLABR2010a.

Figure S1 shows the results. They consist of distributions resulting from Monte-Carlo simulations, where each entry represents one ratio between the steady-state values of *X*_1_ in two scenarios. The top panel shows the ratios under scenario 2 over scenario 1. These ratios are distributed strictly to the right side of zero (in log_10_ space), demonstrating that the new steady state of *X*_1_ in scenario 2 is always as high or higher than the one in scenario 1, no matter what the simulation setting is. Indeed, the ratios of new steady state of X_1_ in scenario 2 differ up to ~2.5 fold (10^0.4^≈2.5) over the corresponding steady state in scenario 1.

A similar result holds when we compare the ratios of new steady-state values of *X*_1_ under scenario 2 versus scenario 3 (bottom panel): Scenario 2 always leads to as strong or stronger responses than scenario 3. Combined, the results in top and bottom panels suggest that, in the presence of a persistent signal *S*_1_, recycling via dephosphorylation of p47^phox^ (scenario 2) allows stronger responses than the other two recycling mechanisms (scenario 1 and 3). Between scenarios 1 and 3, the effectiveness of response is not much different (center panel of Figure S1).


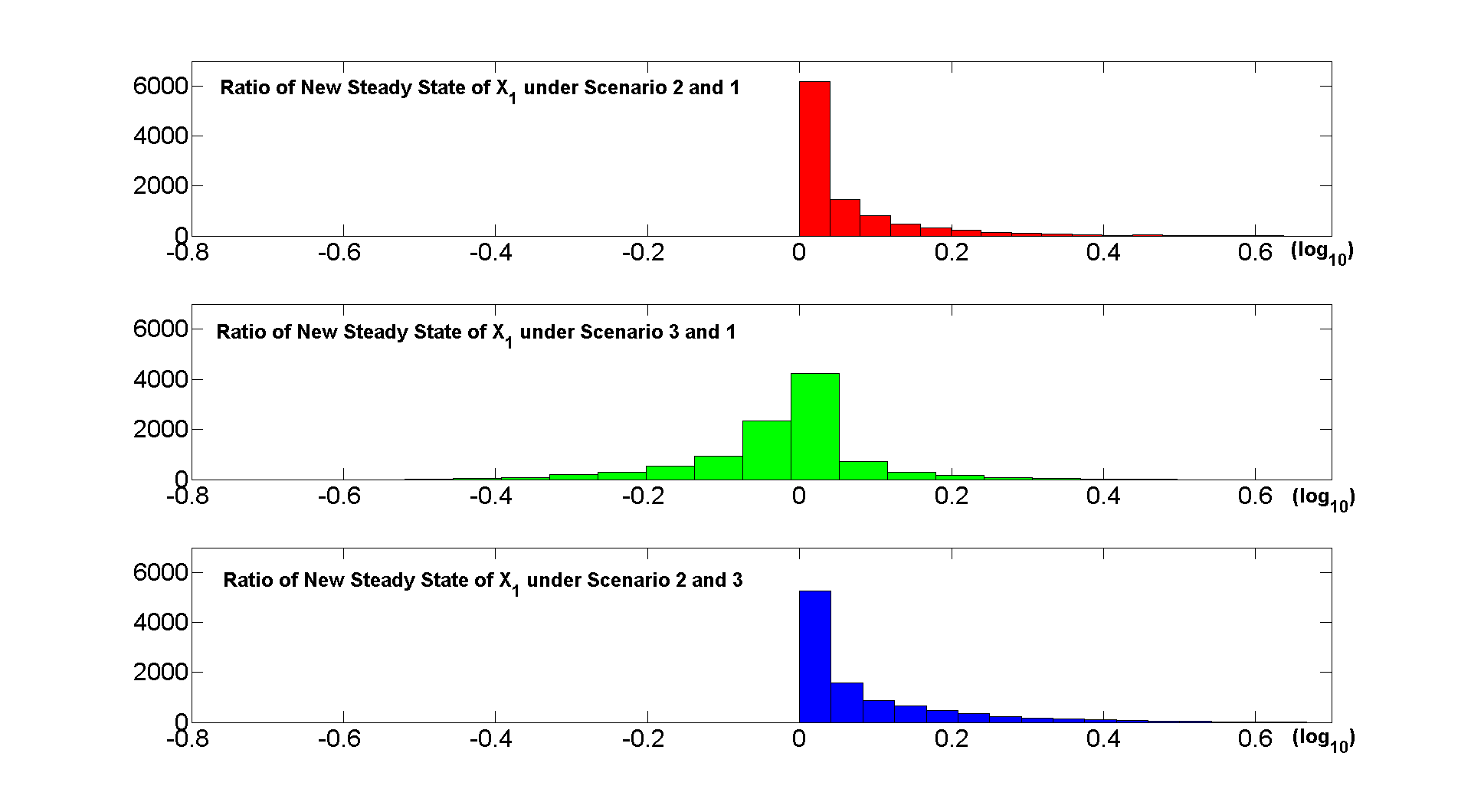


**Figure S1. Simulated responses of the Nox1 system to persistent signals of type *S*_1_ under three extreme recycling mechanisms.** Scenario 1: Only the recycling pathway associated with phosphorylation of NoxA1 (marked as *f*_2_ in Figure1) exists; Scenario 2: Only the recycling pathway associated with dephosphorylation of p47^phox^ (marked as *f*_3_) exists; Scenario 3: Only the recycling pathway associated with deactivation of Rac1_GTP_ (marked as *f*_4_) exists. The horizontal axis indicates the ratio, in log_10_ space, of new steady-state values of *X*_1_ under two different scenarios. For instance, the top panel shows the ratios of *X*_1_ values in Scenario 2 in comparison to Scenario 1 in 10,000 Monte-Carlo simulations. See text for further details.

The example demonstrates that the effectiveness of a particular recycling pathway is interrelated with the demands of the system. For the specific case of a persistent signal *S*_1_, which was studied here, the most efficient recycling mechanism appears to be the dephosphorylation of p47^phox^ (scenario 2). This deduction becomes even stronger when the cell has the option of selecting (or discarding) certain combinations of numerical settings for the rate constants, which in the results in Figure S3 are based on random sampling. For persistent signals of type *S*_2_, *S*_3_, combinations of these signals, or other types of signal trains, the optimal recycling strategy is certainly different. Thus, under the assumption that the cell optimizes responses, one should expect that the cell has developed regulatory mechanisms that control the distribution of recycling fluxes, depending on signaling demands.

***5. Supplementary figures, cited in the text***


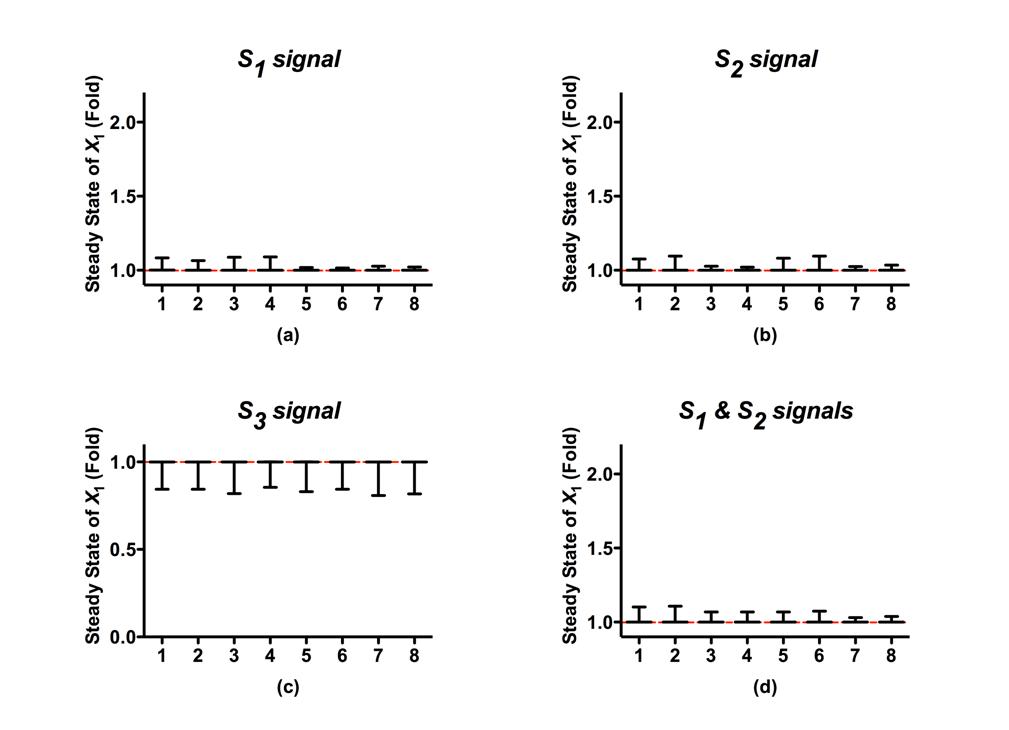


**Figure S2. Monte-Carlo simulation results for evaluating the functional effectiveness of the Nox1 disassembly system against generic criteria of responsiveness.** The four panels show results of the system receiving: (a) signal *S*_1_; (b) signal *S*_2_; (c) signal *S*_3_; (d) both signals, *S*_1_ and *S*_2_. All independent fluxes are sampled from the same uniform distribution (using *U*(0, 2) in log_10_ space). Simulation results are shown by box-and-whisker (min-to-max) plots. The dashed red lines indicate the steady state levels of *X*_1_ under control conditions, which are always set as “1”. Numbers along the *x*-axis correspond to indices in Table 2 and represent different initial conditions. The sample size for each bar is 2,000 points. Details of simulation settings are given in Table S2. See text for further information.


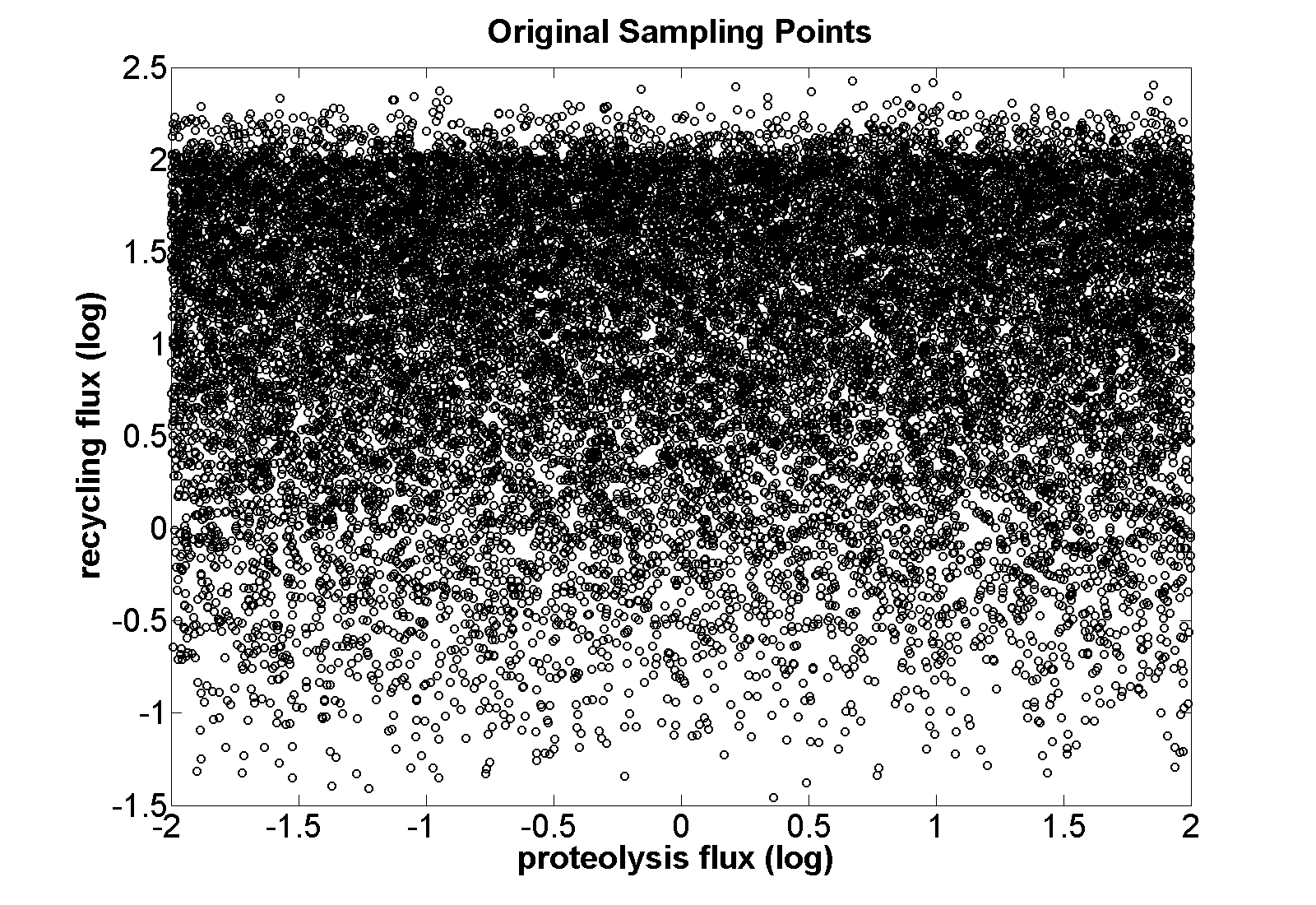


**Figure S3. Original result of a Monte Carlo simulation based on the criterion of the second type.** Sample size: 20,000 points. Values of all independent fluxes were sampled from *U*(0.01, 100). See text for further information.


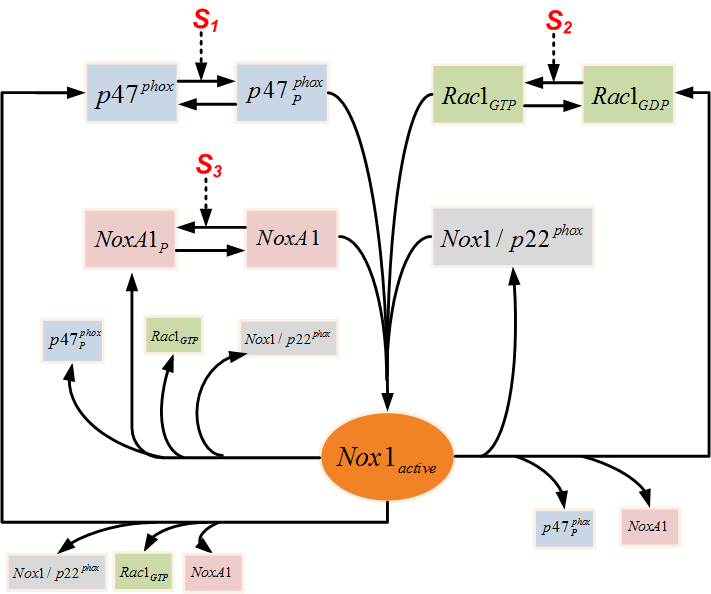


**Figure S4. Schematic representation of the closed Nox1 system.**

***6. Parameter sensitivity analysis***

The proposed Monte-Carlo method allows us to explore distribution ranges, rather than unique specifications, of parameter values. While no hard information regarding these ranges is available, the focus on the *relative* process rates greatly simplifies the analysis, for the following reason. Consider a generic system description in Generalized Mass Action format, as we used it in our analysis:

The speed of its dynamics is determined by the rate constant parameters *γ_ij_*, and multiplication of these rate constants with a common factor *α* (>0) changes the speed of all transients in the same manner; in particular, it does not affect the steady state. Thus, the relative sizes of the *γ_ij_* determine the shapes of the transients, although on an unspecified time scale (see Figure S5). This observation implies that, while we do not know absolute values, the distribution range we specified (10^-4^~10^4^, see Text for detailed explanations) should be sufficiently wide to characterize the relative magnitudes of the rate constant parameters. In other words, the analysis may be performed with relative parameter values, without loss of generality.


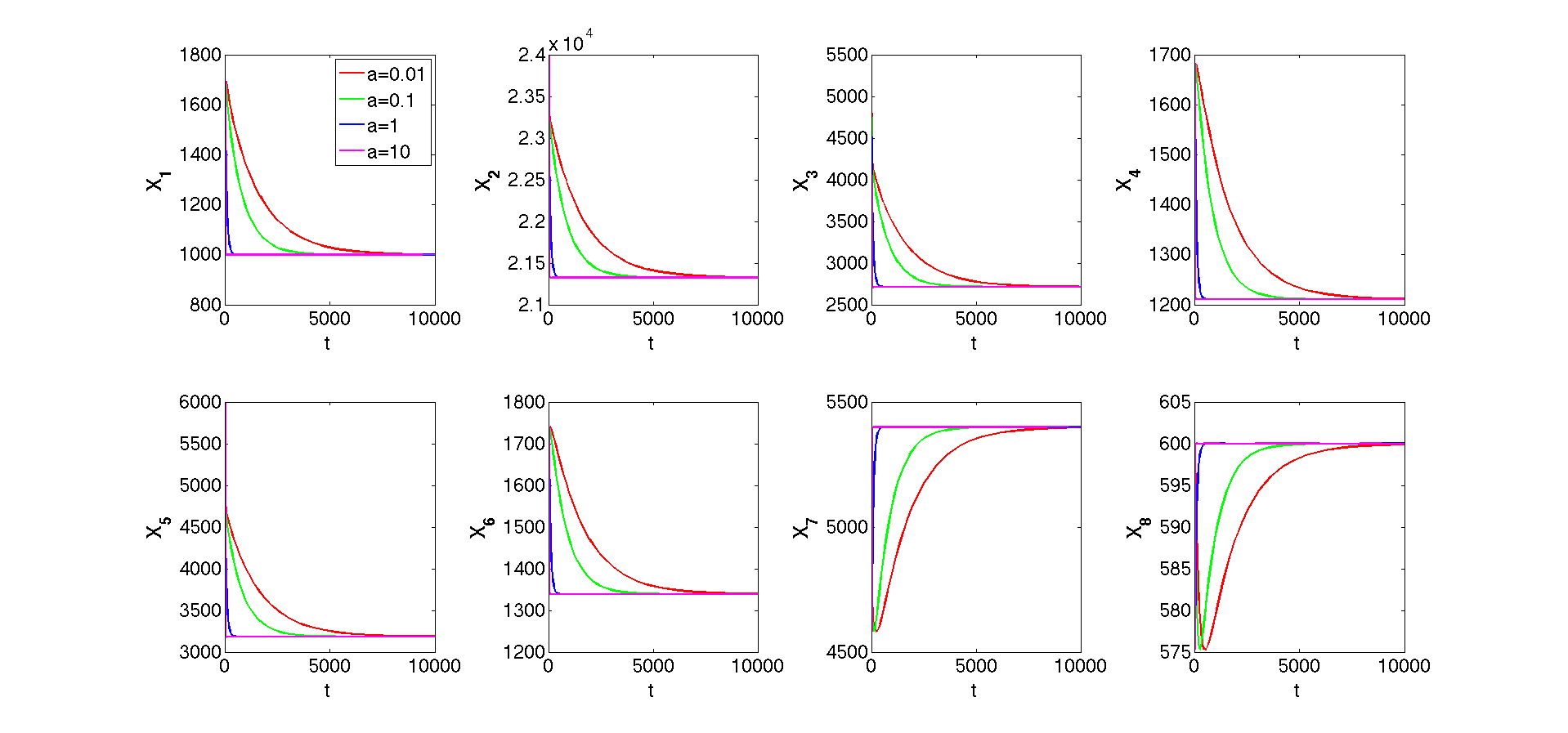


**Figure S5. Simulated system dynamics for different values of a common multiplier *α* of all rate constants.** The multiplier changes the time scale, but not the shapes of the transients.

In order to assess the robustness of the parameters (either independent fluxes or rate constants), extensive sensitivity analyses were performed. The relative sensitivity coefficient (RSC) of a dependent variable *X_i_* with respect to a parameter *γ_j_* is defined as

$S(X_{i},\gamma_{j})=\frac{\partial\ln X_{i}}{\partial\ln\gamma_{j}}=\frac{\partial X_{i}}{\partial\gamma_{j}}\cdot\frac{\gamma_{j}}{X_{i}}\approx\frac{\Delta X_{i}/X_{i}}{\Delta\gamma_{j}/\gamma_{j}}$.

It reflects the relative (infinitesimal) change in a dependent variable *X_i_* (approximated by Δ*X_i_* /*X_i_*) caused by a relative (infinitesimal) change in a parameter γ*_j_* (approximated by Δ*γ_j_* /*γ_j_*), which is assumed to be small. In this setting, an RSC value of 5 indicates that the steady-state concentration of the metabolite *X_i_* is expected to increase by approximately 5% if the parameter *γ_j_* is increased by 1%. Generally, the larger the magnitude of an RSC value, the more sensitive the steady state of *X_i_* is with respect to changes in parameter *γ_j_*. RSC values less than 1 indicate attenuation of perturbations and robustness of the system.

Using the simulation settings described in Section 3 of this Additional File 1, the RSC values of all investigated parameters, namely the independent fluxes *f*_2SS_, *f*_3SS_, *f*_4SS_, *f*_6SS_, *f*_8SS_, *f*_9SS_, *f*_14SS_, and *f*_16SS_ (or equivalently the corresponding rate constants *γ*_2_, *γ*_3_, *γ*_4_, *γ*_6_, *γ*_8_, *γ*_9_, *γ*_14_, and *γ*_16_), with respect to all dependent variables (*X*_1_ to *X*_8_) under the treatment of AngII were calculated using finite differences, and the results are shown in Figure S6-S13. Although the fluxes were randomly selected from a wide range between 0.01 and 100 (using the uniform distribution *U*(-2, 2) in log_10_ space, as we did in the article), the computed RSC values are consistently small and less than 1 in magnitude. This result indicates that small changes in the investigated parameters will result in even smaller changes in the steady states of all dependent variables, thereby strongly demonstrating the insensitivity and robustness of the system. Similar sensitivity results were obtained by using the simulation settings described in Section 2 of this Additional File 1 (data not shown), which further confirms the robustness of the parameters.


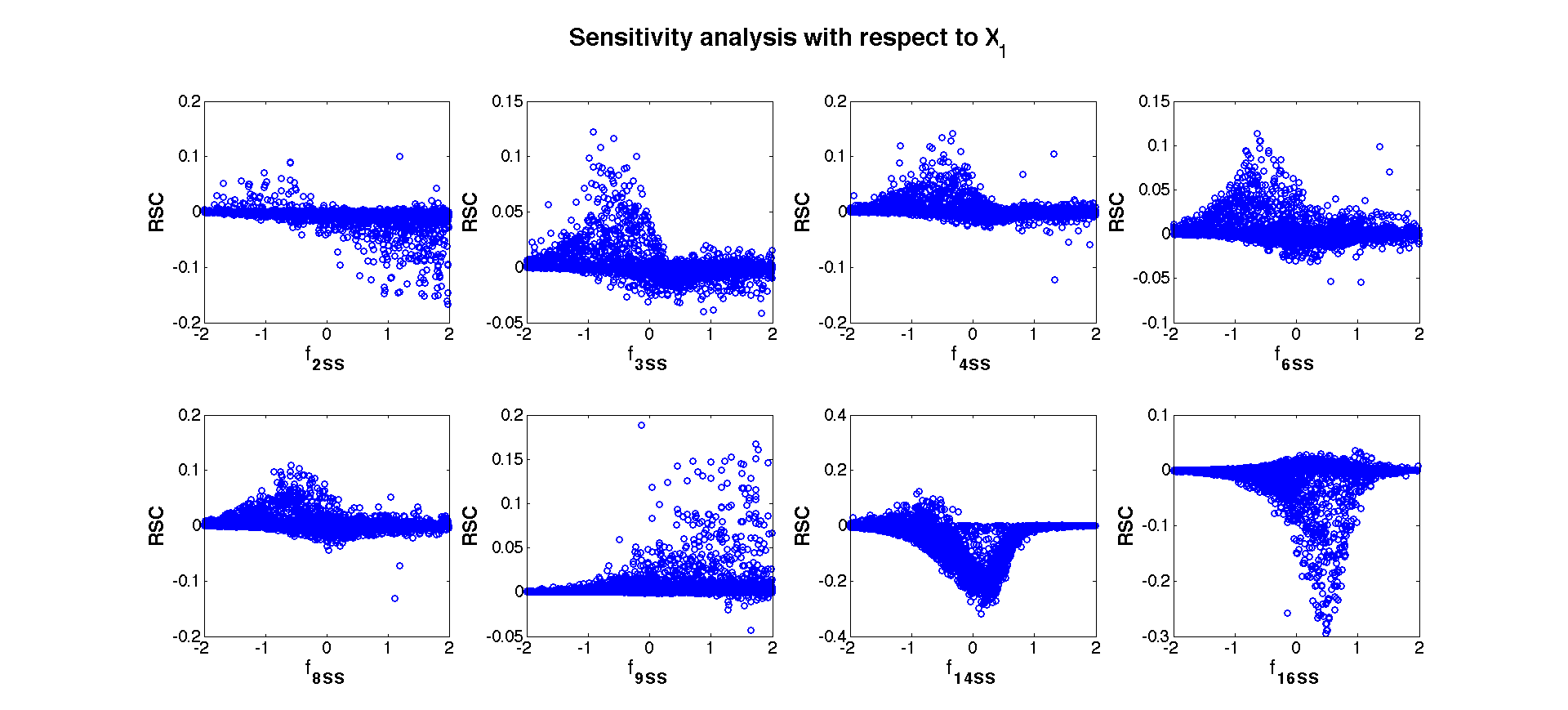


**Figure S6. Sensitivity analysis with respect *X*_1_.** The *y*-axis represents the relative sensitivity coefficient (RSC) and the *x*-axis represents the sampled independent fluxes, which are expressed in log_10_ space.


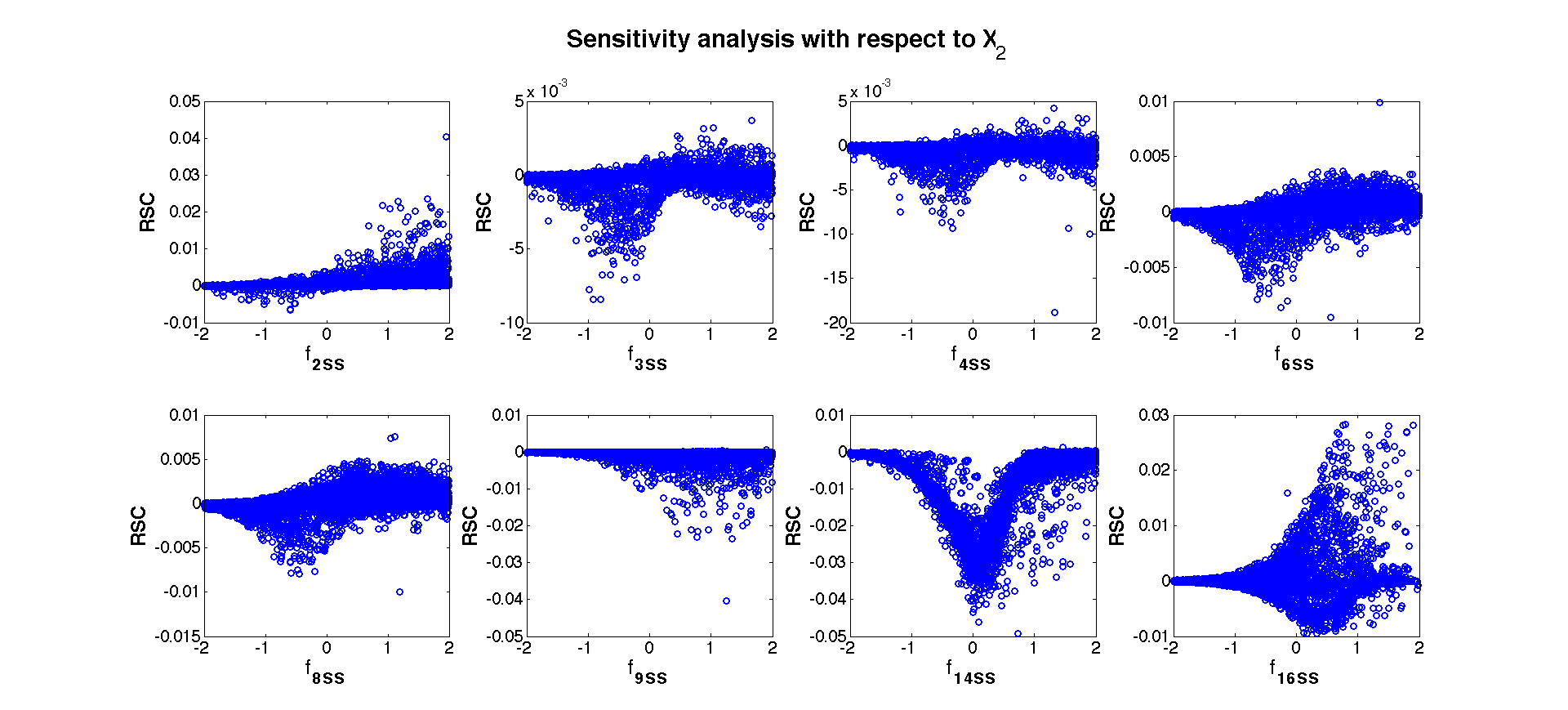


**Figure S7. Sensitivity analysis with respect *X*_2_.** The *y*-axis represents the relative sensitivity coefficient (RSC) and the *x*-axis represents the sampled independent fluxes, which are expressed in log_10_ space.


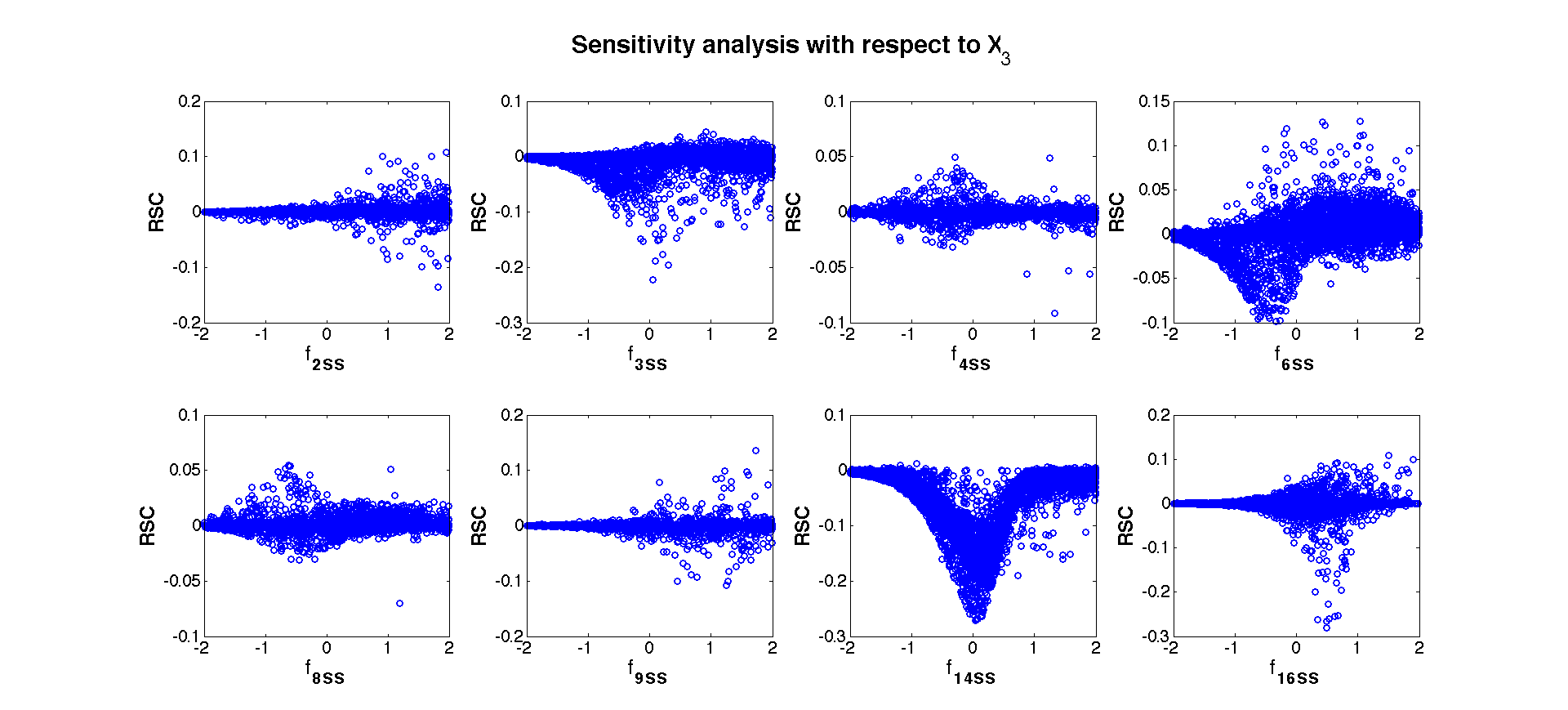


**Figure S8. Sensitivity analysis with respect *X*_3_.** The *y*-axis represents the relative sensitivity coefficient (RSC) and the *x*-axis represents the sampled independent fluxes, which are expressed in log_10_ space.


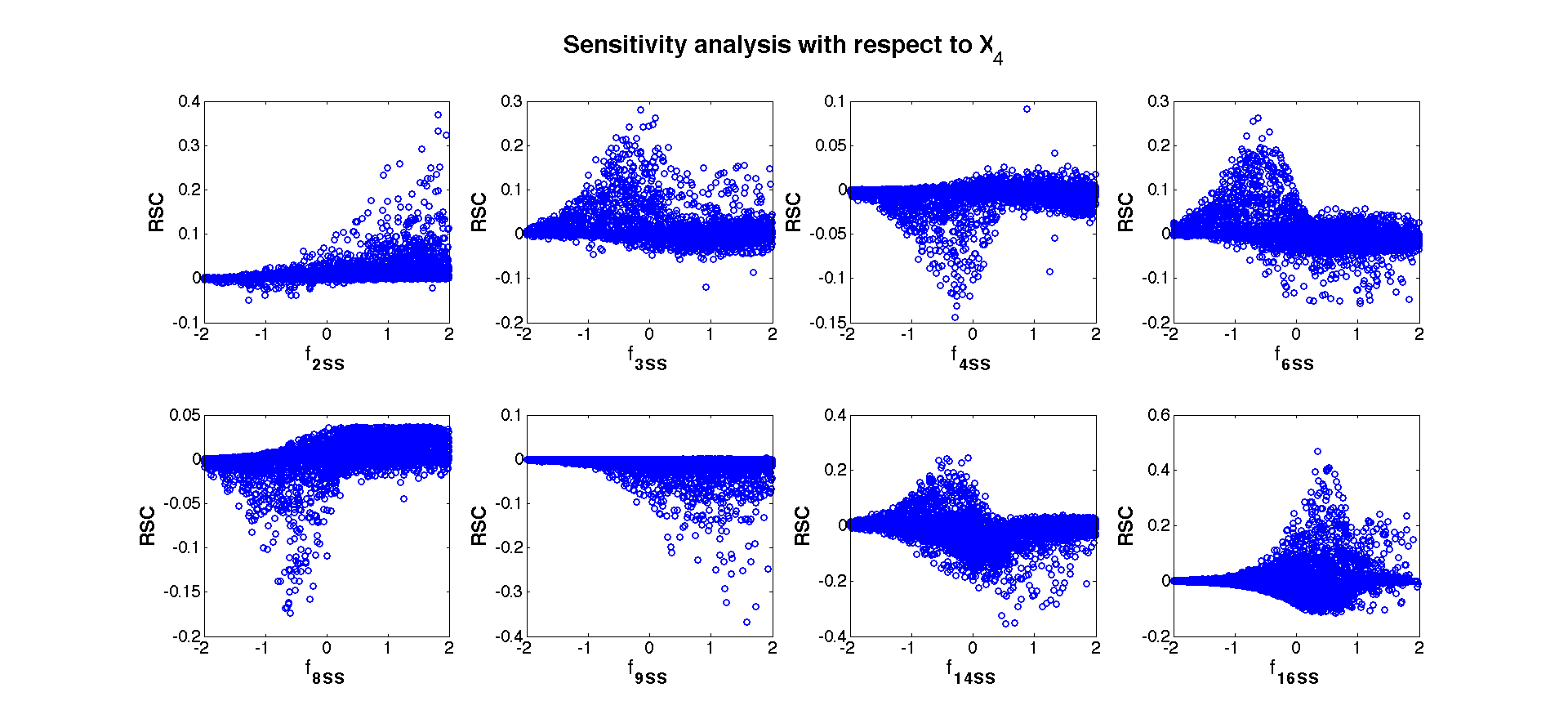


**Figure S9. Sensitivity analysis with respect *X*_4_.** The *y*-axis represents the relative sensitivity coefficient (RSC) and the *x*-axis represents the sampled independent fluxes, which are expressed in log_10_ space.


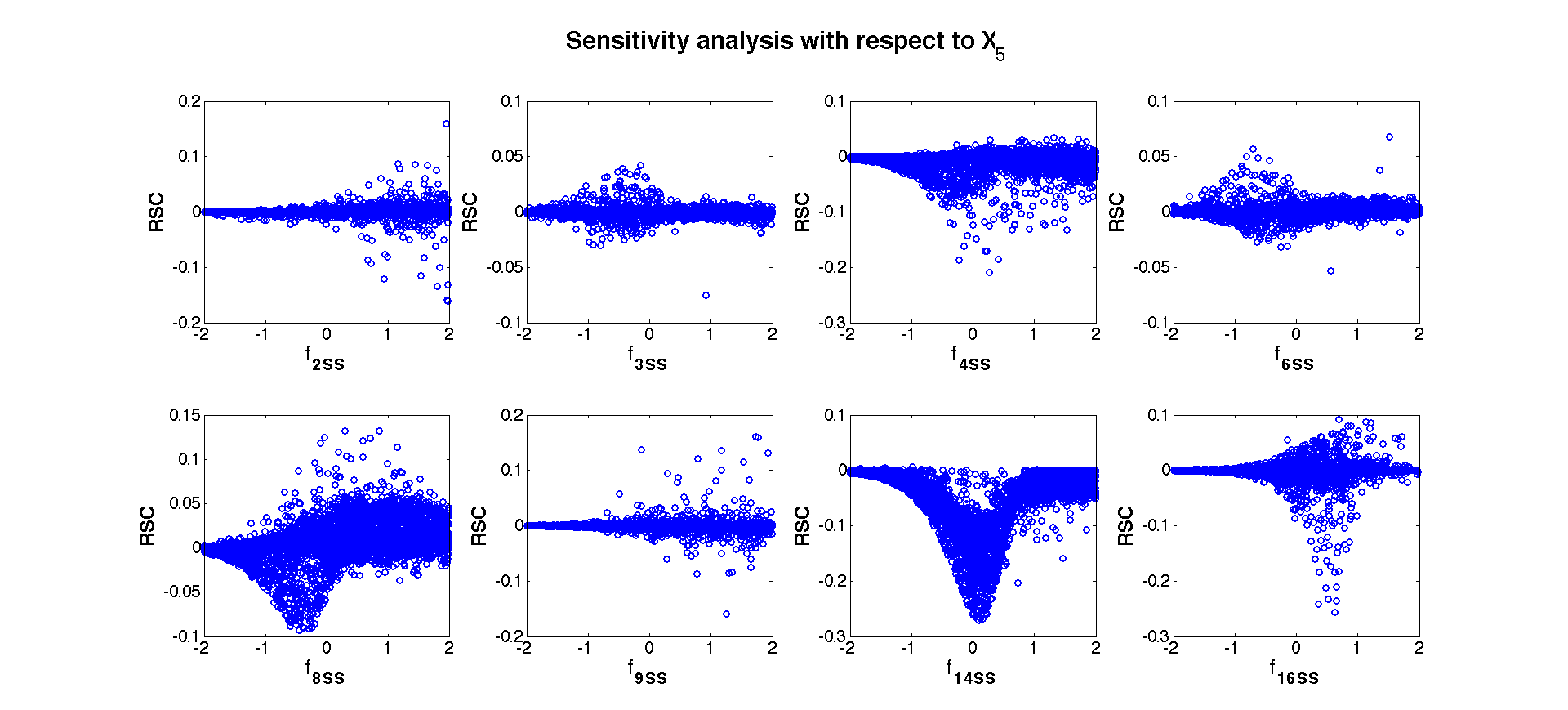


**Figure S10. Sensitivity analysis with respect *X*_5_.** The *y*-axis represents the relative sensitivity coefficient (RSC) and the *x*-axis represents the sampled independent fluxes, which are expressed in log_10_ space.


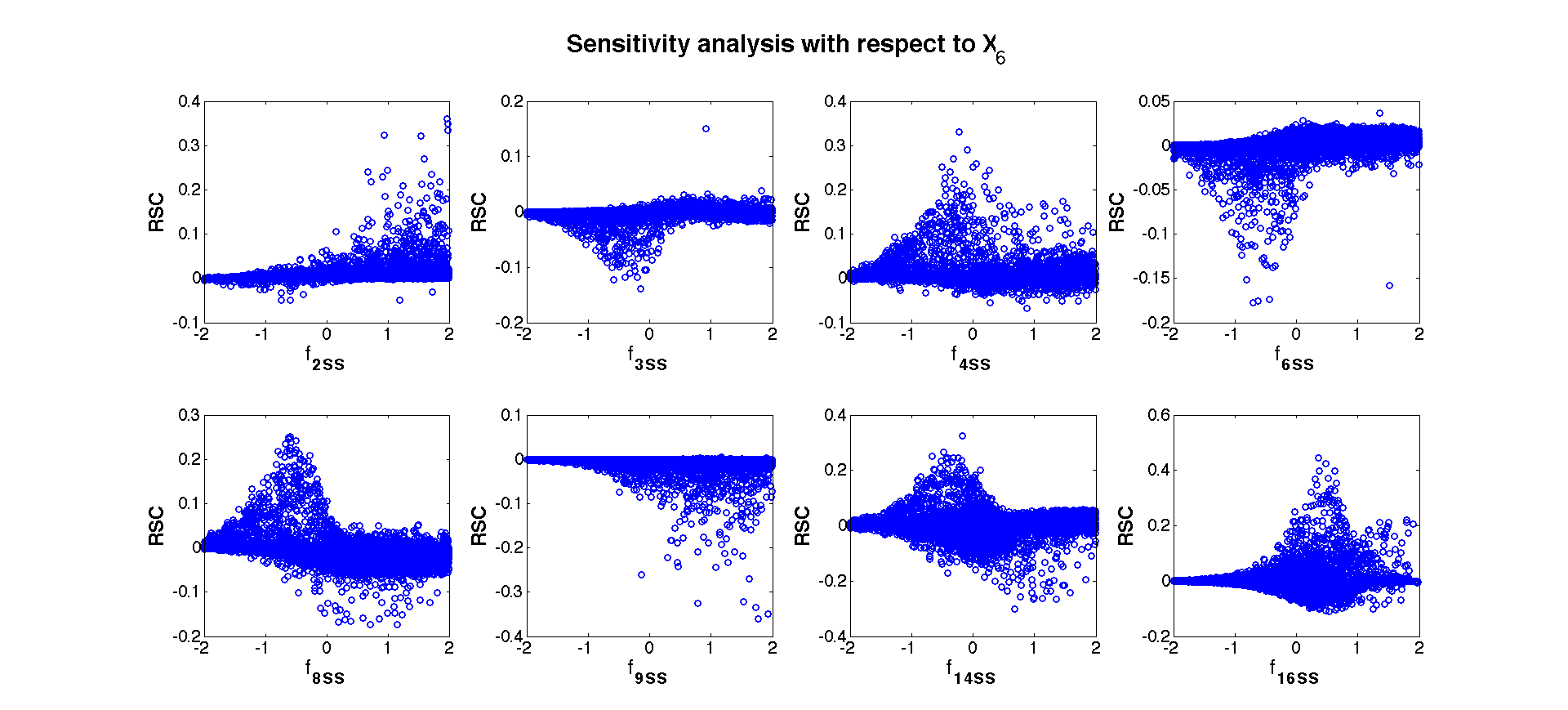


**Figure S11. Sensitivity analysis with respect *X*_6_.** The *y*-axis represents the relative sensitivity coefficient (RSC) and the *x*-axis represents the sampled independent fluxes, which are expressed in log_10_ space.


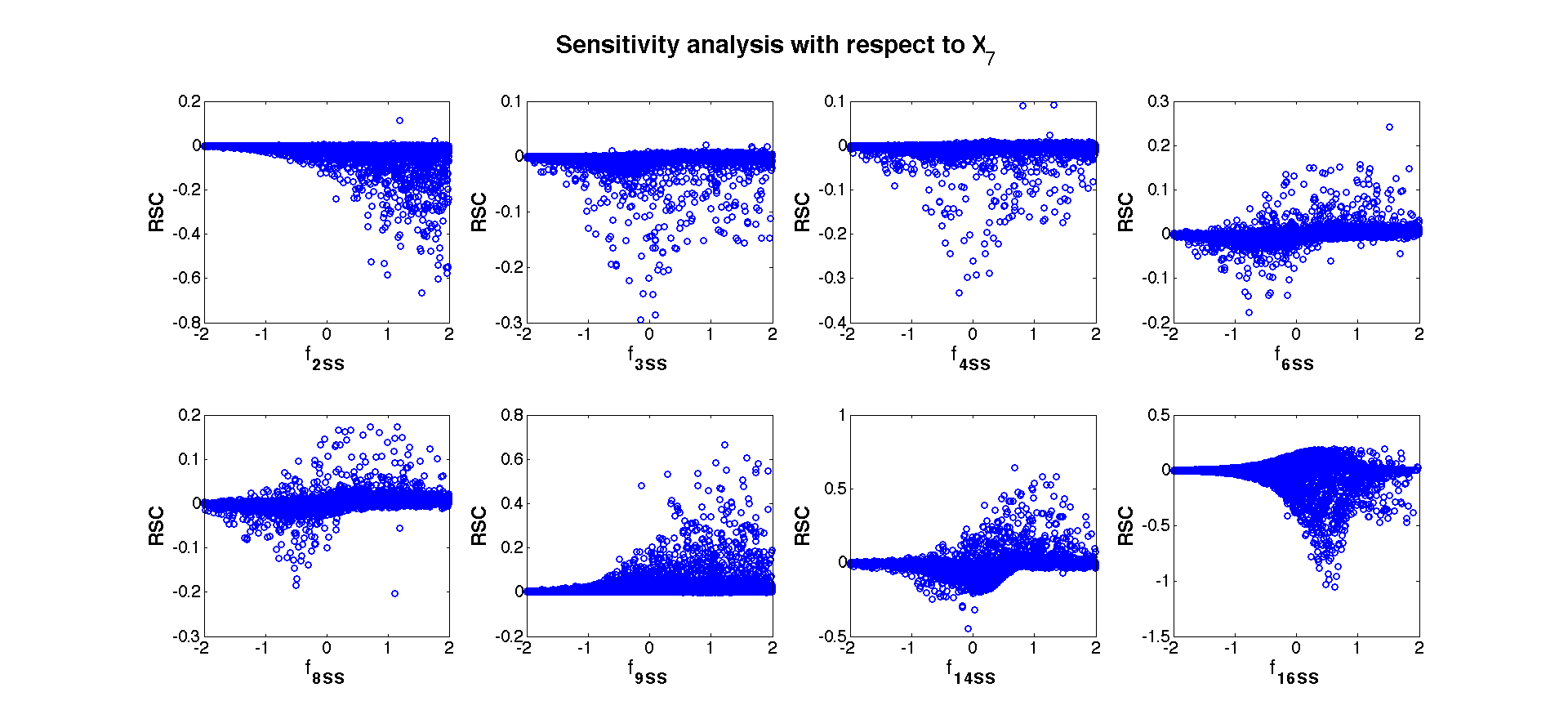


**Figure S12. Sensitivity analysis with respect *X*_7_.** The *y*-axis represents the relative sensitivity coefficient (RSC) and the *x*-axis represents the sampled independent fluxes, which are expressed in log_10_ space.


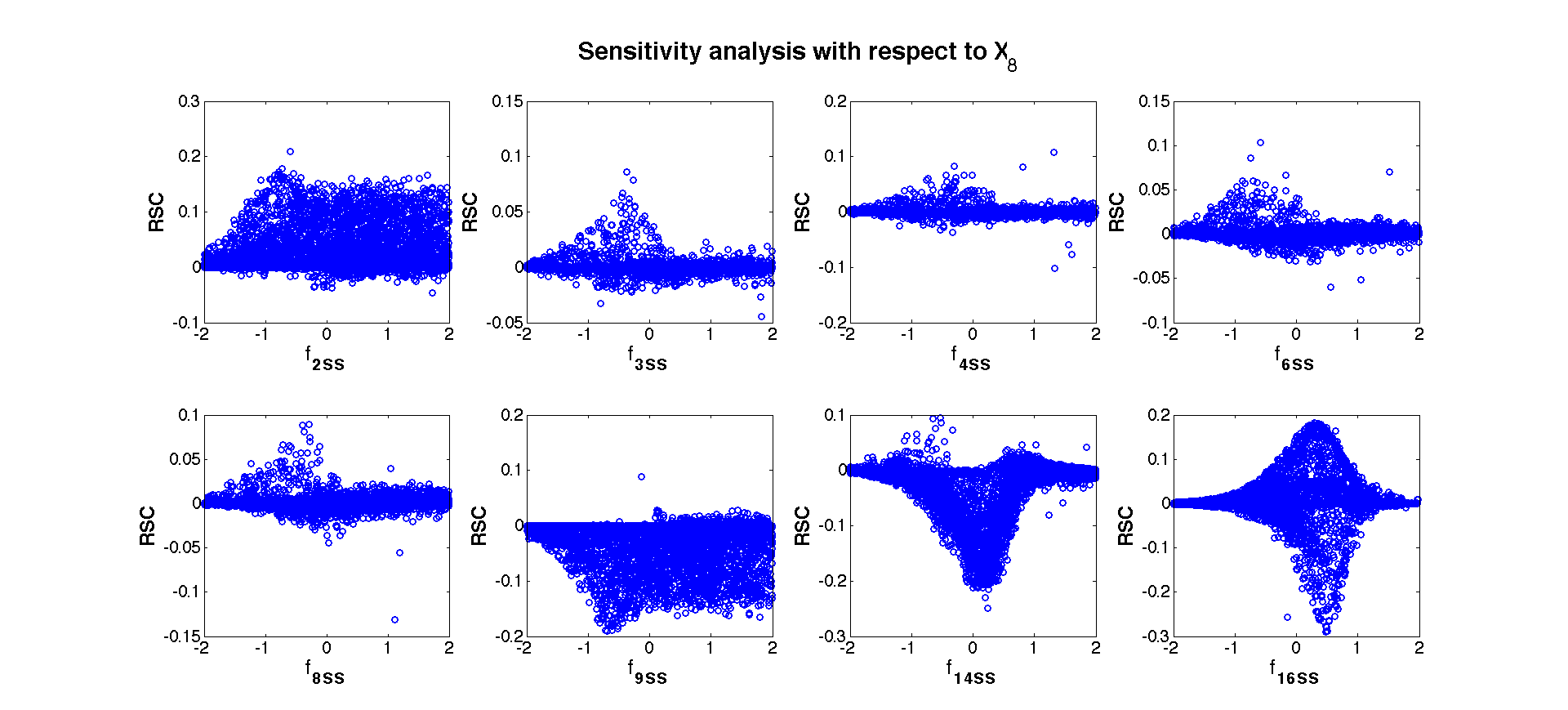


**Figure S13. Sensitivity analysis with respect *X*_8_.** The *y*-axis represents the relative sensitivity coefficient (RSC) and the *x*-axis represents the sampled independent fluxes, which are expressed in log_10_ space.
